# New Retinal Pigment Epithelial Cell Model to Unravel Neuroprotection Sensors of Neurodegeneration in Retinal Disease

**DOI:** 10.3389/fnins.2022.926629

**Published:** 2022-06-30

**Authors:** Aram Asatryan, Jorgelina M. Calandria, Marie-Audrey I. Kautzmann, Bokkyoo Jun, William C. Gordon, Khanh V. Do, Surjyadipta Bhattacharjee, Thang L. Pham, Vicente Bermúdez, Melina Valeria Mateos, Jessica Heap, Nicolas G. Bazan

**Affiliations:** Neuroscience Center of Excellence, School of Medicine, Louisiana State University Health New Orleans, New Orleans, LA, United States

**Keywords:** neuroprotectin D1, RPE cell, single cell, gene expression, lipids, apoptosis, autophagy, age-related macular degeneration (AMD)

## Abstract

Retinal pigment epithelial (RPE) cells sustain photoreceptor integrity, and when this function is disrupted, retinal degenerations ensue. Herein, we characterize a new cell line from human RPE that we termed *ABC*. These cells remarkably recapitulate human eye native cells. Distinctive from other epithelia, RPE cells originate from the neural crest and follow a neural development but are terminally differentiated into “epithelial” type, thus sharing characteristics with their neuronal lineages counterparts. Additionally, they form microvilli, tight junctions, and honeycomb packing and express distinctive markers. In these cells, outer segment phagocytosis, phagolysosome fate, phospholipid metabolism, and lipid mediator release can be studied. ABC cells display higher resistance to oxidative stress and are protected from senescence through mTOR inhibition, making them more stable in culture. The cells are responsive to Neuroprotectin D1 (NPD1), which downregulates inflammasomes and upregulates antioxidant and anti-inflammatory genes. ABC gene expression profile displays close proximity to native RPE lineage, making them a reliable cell system to unravel signaling in uncompensated oxidative stress (UOS) and retinal degenerative disease to define neuroprotection sites.

## Introduction

The retinal pigment epithelial (RPE) cells conform to a tightly arranged monolayer that serves as a barrier between the photoreceptor cells (PRC) and the choriocapillaris (Bazan, 2007; Lakkaraju et al., 2020). In addition, these cells are critical in nutrient transport, efflux of catabolic products, and the daily phagocytosis of PRC outer segments. The RPE also recycles all-trans-retinal that is oxidized during photo conversion and docosahexaenoic acid (DHA) through the interphotoreceptor matrix (IPM) to the base of inner segments and secretes cytokines, chemokines that locally modulate innate and adaptive immune systems (Bazan et al., 1985; Gordon et al., 1992; Detrick and Hooks, 2020; Lakkaraju et al., 2020; Storm et al., 2020).

Retinal pigment epithelial cells and PRC are at constant risk for uncompensated oxidative stress (UOS) because of their oxygen-rich environment, high flux of polyunsaturated fatty acids (PUFAs) (Bazan et al., 2010), and high metabolic activity (Bazan, 2006, 2007). RPE cell impairments due to disruption in homeostasis are involved in retinal degenerative diseases, including age-related macular degeneration (AMD), where perturbed phagocytic activity occurs (Mitter et al., 2014; Golestaneh et al., 2017; Inana et al., 2018).

Three phagocytic processes are at crossroads with autophagy in RPE. Daily phagocytosis of the PRC tips engages the microtubule-associated protein 1 light chain 3 (LC3) in LC3-associated phagocytosis (LAP), and autophagy at the basal level requires LC3 for the formation of the phagophores as part of the normal repair process, which includes mitophagy that eliminates damaged mitochondria by oxidative stress (Intartaglia et al., 2021). Downstream, autophagic lysosome-mediated degradation is often positively regulated by AMP-activated protein kinase (AMPK) signaling and negatively regulated by the mammalian/mechanistic target of rapamycin (mTOR) pathway (He and Klionsky, 2009). AMPK regulates lipid metabolism and cell survival via adiponectin and its receptors, Adipor1 and Adipor2, by stimulating ceramidase activity. Previously, we have demonstrated that Adipor1 mutant mice, independent of their cognate ligand adiponectin, result in retinal degeneration (Rice et al., 2015). A single amino acid mutation of *Adipor1* has been found in different forms of retinitis pigmentosa (Xu et al., 2016; Zhang et al., 2016). Membrane frizzled-related protein (MFRP) and AdipoR1 are critical for DHA uptake and retention in the RPE and retina (Rice et al., 2015; Kautzmann et al., 2020). Moreover, a recent study suggests a role for Adipor1 in the pathogenesis of Late-Onset Retinal Degeneration (L-ORD) (Miyagishima et al., 2021). We characterized human primary RPE cells, dubbed ABC, that display features of the native pigment epithelium. Notably, those features include formation of impermeable monolayers with tight junctions and high resistance to UOS. These cells do not undergo senescence and display functional phagocytosis and active lipid dynamics mechanisms. Here we used ABC cells in a case study to unravel the relationship between these mechanisms in the normal cycle of the cell.

## Materials and Methods

### Cell Lines

The ABC cell line was derived from the ocular globes of a 19-year-old male donor provided by the National Disease Research Interchange (NDRI) within 24 h after death (head trauma), following a modification of a previously described protocol by Ishida et al., 1998 (Calandria et al., 2012). After removing the vitreous humor, flaps of the retina, choroid, and RPE were made, and squares of 5 mm × 5 mm were cut and placed in Petri dishes. The RPE cells grew for 3 weeks in the dishes, then were transferred to flasks with Minimum Essential Medium Eagle (MEM, Millipore Sigma, Burlington, MA, United States, Cat# M2279) containing 10% fetal calf serum, 5% newborn calf serum, 1X non-essential amino acids, 4 mM glutamine, amphotericin B (0.5 g/ml), and gentamicin (10 μg/ml). The cells were then trypsinized (0.05%); dead and loosely attached cells were washed and discarded. The remainder of the attached cells was further trypsinized (0.25% trypsin) and passaged several times until a homogeneous culture was obtained. ARPE-19 cells were obtained from ATCC, Manassas, Virginia, Cat# CRL-2302 and cultured as recommended by the commercial vendor. Additionally, primary human RPE49 cell line was developed by isolation of the RPE from a healthy 49-year-old male Caucasian donor and cultured in the same conditions as the ABC cells.

### Basal Flux Measurement

ABC cells were grown in Thincert cell culture inserts (Greiner Bio-One, Monroe, NC, United States) with permeable polystyrene membranes of 12 mm diameter and 0.4 mm pore size previously treated with Vitronectin. Basal (lower) and apical (upper) compartments were loaded with 1.5 and 0.5 ml of medium, respectively, for equilibrium. After 7 days of culture, the impermeability of confluent RPE cells was tested by adding a medium containing 0.04% trypan blue (1 ml) to the upper compartment, producing an apical-to-basal hydrostatic pressure. Then cell cultures were incubated under these conditions for up to 12 h. Empty well inserts were used as controls. The basal flux was calculated by photometry measurements after subtracting the background and is expressed in absorbance at 560 nm. After trypan blue treatment, cells were washed with medium and subsequently stained with Alexa Fluor 594 WGA (Invitrogen, Waltham, MA, United States, Cat# W11262) and Hoechst for highly selective staining of the plasma membrane and nucleus, respectively. Control inserts without cells were compared with inserts containing ABC cells.

### Cell Culture, Staining, and Morphology

The cells were routinely passaged by dissociation in 0.05% (w/v) trypsin in MEM medium, followed by replating at a split ratio ranging from 1:3 to 1:6 (Sonoda et al., 2009). ABC cells were maintained in T75 flasks in the medium described below. Primary cultures were incubated in the culture medium [MEM medium containing 10% FBS, 5% NCS, MEM-NEAA (Gibco, Carlsbad, CA, United States, Cat# 11140050)] and 1× Penicillin/Streptomycin at 37°C, 5% CO_2_, and 99% relative humidity for 2 days. The medium was replaced weekly. Cells of passage numbers 20–25 were used in all of the experiments. Proliferation of cells was determined using anti-Ki67 antibody (rabbit polyclonal, Abcam Cat# ab15580) as described (Al-Hussaini et al., 2008). Briefly, ABC cells were seeded at 250,000 cells/ml in 12-well plates and incubated overnight for 2, 3, 4, 5, 6, 7, and 8 days to reach several degrees of confluency, then they were fixed with 4% paraformaldehyde and immunostained with anti-Ki67 (Millipore Sigma, Burlington, MA, United States, Milli-Mark Anti-Ki67, clone Ki-S5 APC conjugate Cat# FCMAB103AP). Hoechst 33342 counterstain was included to measure apoptosis as previously described (Calandria et al., 2015). To induce UOS, cells were serum-starved for 8 h and exposed to H_2_O_2_ (1,600 μM)/TNFα (10 ng/ml) for 24 h. After that period, cells were photographed with a DIAPHOT 200 microscope (Nikon, Melville, NY, United States) with fluorescent optics. Images were recorded by a color-chilled 3CCD camera (Hamamatsu, Bridgewater, NJ, United States) and counted with ImageJ^1^ using small-size/high-brightness to detect apoptotic cells with the blue filter, and small red dots in the nuclei on the red filter were counted as positive Ki67 cells. To assess the morphology of ABC cells, they were grown on nitrocellulose membranes for 1 week or pelleted, then fixed and embedded in plastic and thin-sectioned as described (Knott et al., 2011).

### Quantitative PCR and Real-Time PCR

RNA was collected at passages from ABC cells (P# 18 and 21), ARPE-19 cells (P# 22), and primary human RPE49 (isolated from a healthy 49-year-old donor). One μg of total RNA was reverse transcribed using an iScript cDNA Synthesis Kit (Bio-Rad, Hercules, CA, United States, Cat# 1708890). Quantitative PCR (qPCR) was performed in a CFX-384 Real-Time PCR system (Bio-Rad) using primers (Supplementary Table 1), and then amplification products were loaded on 2% agarose gels (Supplementary Figure 1A). Data were normalized using Delta-Delta-Ct (ΔΔCt). All results are expressed as means ± SEM, *n* = 4 (Supplementary Figure 1B).

### Western Blot

Samples were lysed by RIPA buffer, and protein was determined by Bradford assay (Bio-Rad). After denaturation, 30 μg of total protein for cell samples were separated by SDS-PAGE (4–12% gradient) gel (Thermo Fisher Scientific, Waltham, MA, United States) and transferred to nitrocellulose membranes (Bio-Rad). The membranes were blocked by 5% non-fat dry milk in PBST, probed with anti-Bestrophin (Abcam, Cat# ab2182), anti-Cytokeratin 8 (Santa Cruz Biotechnology, Dallas, TX, United States, Cat# sc-8020), anti-rhodopsin (Abcam, Cambridge, United Kingdom, Cat# ab81702), anti-GAPDH (Millipore Sigma, Burlington, MA, United States, Cat# MAB374) for 1 h, washed three times with PBST, probed with secondary antibodies (GE Healthcare, Chicago, IL, United States) for 1 h, and washed three times with PBST. Protein bands were visualized using the LAS 4000 imaging system (GE Healthcare).

### Uncompensated Oxidative Stress Induction and Lipid Treatments

Cells were grown in 6-well plates semi-confluent for 72 h in DMEM/F12 and 10% fetal bovine serum (FBS) media, then serum starved for 8 h before triggering oxidative stress by further incubation with 10 ng/ml of TNF-α plus 600 μM H_2_O_2_ for 15 h. Bioactivity was assayed by adding 50 nM of neuroprotectin D1 (NPD1) at the outset of oxidative stress.

### Phagocytosis Imaging

Eight-well chamber slides (LabTek # 154534) were seeded with 100,000 cells per well for each cell type and incubated overnight at 37°C and 5% CO_2_. To evaluate the amount of photoreceptor outer segments (POS, bovine rod photoreceptor outer segments, InVision BioResources, Seattle WA, United States, Cat# 98740) particles to add to each well, we followed the protocol described in Mukherjee et al., 2007 and determined that we needed 10 × 10^6^ POS/well. We used fluorescein isothiocyanate (FITC; Invitrogen # F1906) diluted in DMSO at a 10 mg/ml concentration and labeled the POS at a 3:1 ratio (POS vol:FITC vol). The tube containing FITC + POS was shielded from light and incubated on a rotating well for 1 h at room temperature. The labeled POS were rinsed by adding 1 mL of DMEM + 2.5% sucrose to the sample. The tube was spun at 3,000 *g* for 5 min at room temperature, and then the supernatant was removed, rinsed two additional times, and transferred into a fresh tube. Before distribution into the wells, the labeled POS were added to the culture medium, and this mixture was added to each well at a 10 × 10^6^ density. Yellow-green fluorescent beads (Invitrogen # F13081) at a concentration of 10 × 10^6^ beads/μl were distributed at a density of 10 × 10^6^ beads/well after mixing with culture medium. Cells were incubated at 37°C + 5% CO_2_ and collected at 6, 8, and 22 h following the protocol (Mao and Finnemann, 2013) and stained with Hoechst. Cells were imaged using an LSM 710 Zeiss confocal microscope with a 63X oil-immersed objective. Z-stacks with 0.5 μm step-size were acquired in four different areas of the well for each experimental time point. Maximal projections of the z-stacks were captured and exported in ImageJ (see footnote 1) for fluorescence intensity measurements.

### Evaluation of POS Phagocytosis by Flow Cytometry

To assess phagocytosis, ABC cells were either plated on Thincerts™ cell culture inserts coated with Vitronectin or plated in an 8-well chamber slide and grown for 3 days to 1 week. Cells were serum starved and then fed with POS for 8 h at a 1:10 ratio (cell:POS). Blue-fluorescent Hoechst 33342 dye (1:500) was from Invitrogen (Carlsbad, CA, United States). Cell samples were treated and then analyzed immediately on a Gallios Flow Cytometer (Beckman Coulter). Cells were gated based on the specific set of cell labeling used in each experiment (Figure 4 and Supplementary Figure 4). After excluding proper gating cell debris and doublets for Apoptosis/Necrosis assay (Enzo Life Sciences, Farmingdale, NY, ENZ-51002-25), a plot of Annexin V versus 7AAD signals was generated (Supplementary Figure 4). Twenty thousand or more events were collected per treated sample. Data were analyzed using Kaluza Analysis Software (Beckman Coulter). For each condition for ABC control cells vs. ABC cells exposed to FITC-POS (Figure 4F, top and bottom, respectively), 7AAD versus FITC signals and unbound/internalized POS were obtained (100,000 events analyzed in each condition). Then, after gating out unbound POS, viable (7AAD negative) and apoptotic cells (7AAD positive, gated, right) are revealed. Confluent ABC cell cultures were incubated with previously labeled (pHrodo or FITC) or unlabeled bovine POS (1:10 cell:POS ratio) for 16 h. Then cells were trypsinized and labeled for flow cytometry processing (Supplementary Figure 4).

**FIGURE 1:**
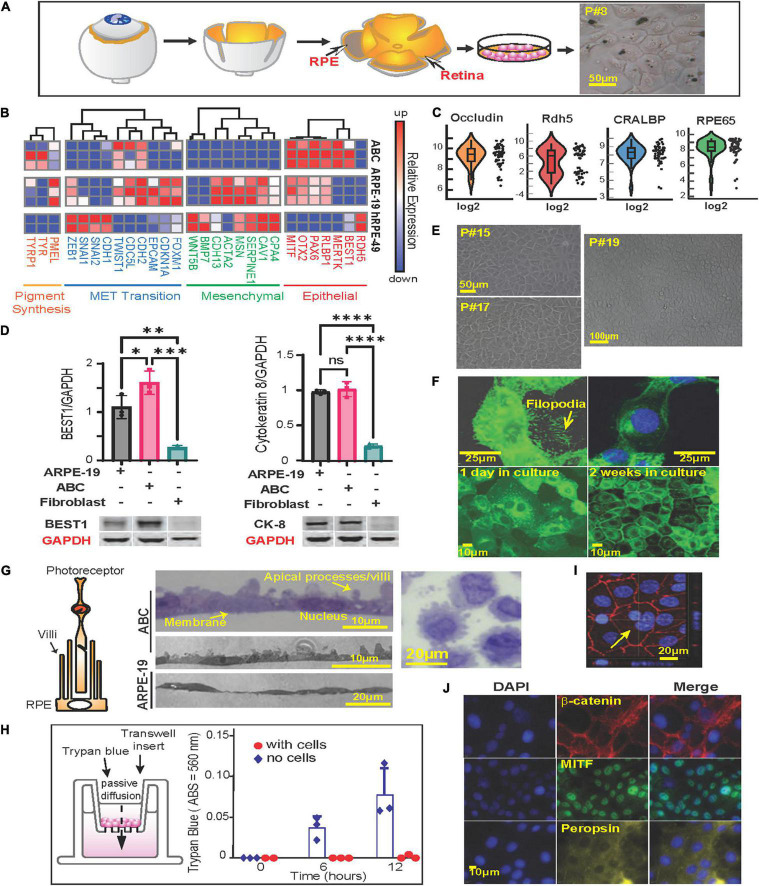
Phenotypycal characteristics of primary human RPE (ABC cells). **(A)** RPE cells were isolated from human donor eyes and grown in tissue culture. Schematic representation of the protocol used for the isolation of RPE cells. Right panel shows light microscopy photograph of passage 8 (P#8) ABC cells with melanosomes. **(B)** Heatmap showing RNA sequencing results comparing ABC, ARPE-19 and human primary RPE cells from a 49-year-old donor (hRPE49) mRNA expression levels for Pigment synthesis, MET Transition signaling, Mesenchymal and Epithelial markers. **(C)** Violin plots of single-cell RT-PCR for ABC cells showing distribution of RPE-specific markers whitin the population. **(D)** Western blot comparison of the levels of Cytokeratin-8, and Bestrophin-1 (BEST1). The bars represent the mean and SEM of 3 independent measurements. GAPDH was used for standardization. One Way ANOVA and *t*-test was applied to determine significance. ^∗^*p* < 0.05; ^∗∗^*p* < 0.01; ^∗∗∗^*p* < 0.001; ^∗∗∗∗^*p* < 0.0001. Representative blots are displayed. The whole membranes are depicted in Supplementary Figure 2. **(E)** ABC cell passages 15, 17, and 19 forming honeycomb like monolayers. **(F)** Slender cytoplasmic projections (filopodium) in migrating ABC cells are shown in GFP-tagged MFRP overexpressing ABC cells. Monolayer formation. **(G)** Representation of RPE cells microvilli surrounding photoreceptor. Sections of confluent RPE cells growing on a nitrocellulose filter. Sections through an RPE cell pellet show cells to be about 20 μm wide and 15 μm thick with prominent lateral protrusions (apical villar processes) and a single large central nucleus, resembling the *in vivo* retinal RPE cell layer. **(H)** ABC cells seeded on Thincert cell culture inserts treated with vitronectin showing the functional junction formation of ABC cells (Supplementary Figure 4B). **(I)** ZO-1 staining of ABC cells in culture, one week after plating. The cells are tightly packed. Yellow arrow shows a double nucleated ABC cell. Basal flux was calculated by photometry measurements after subtracting the background and is expressed in absorbance at 560 nm. **(J)** Immunocytochemistry of ABC cells after 1 week in culture, cells express MITF, beta-catenin, and Peropsin. Blue = DAPI; Red = β-catenin; Green = MITF; Yellow = Peropsin.

**FIGURE 2:**
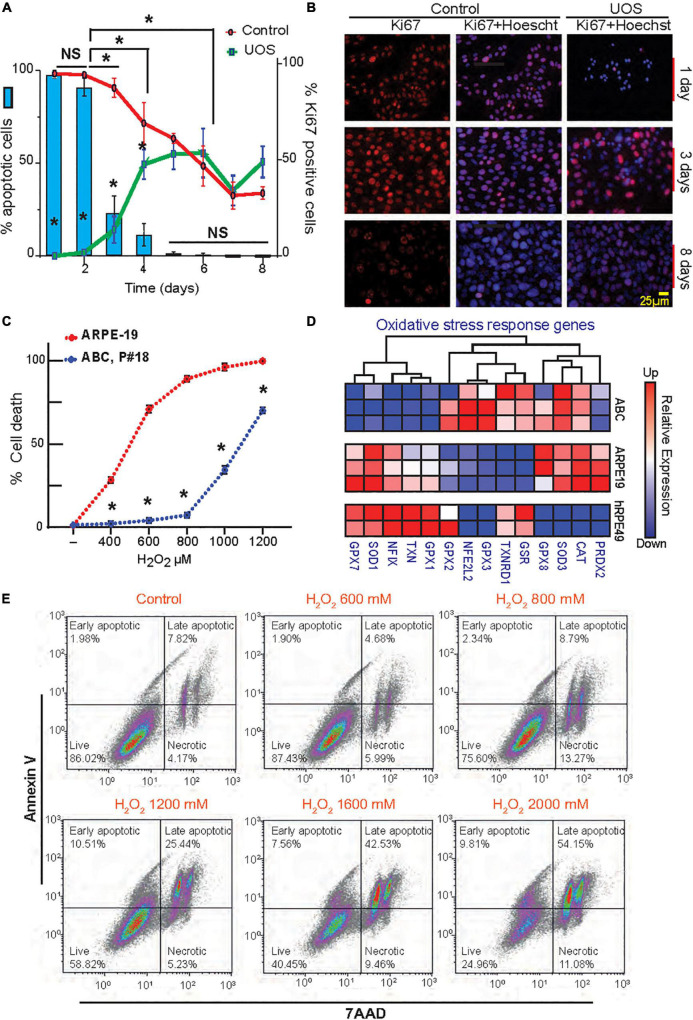
Postmitotic ABC cells become resistant to UOS. **(A)** The expression of Ki67 in ABC cells is represented by green (UOS) and red (control) lines (right y-axis). Histogram shows cell death rate (left y-axis). **(B)** Representative images of Ki67 staining in ABC cells at different times. **(C)** ABC and ARPE-19 cells were exposed to various concentrations of H_2_O_2_. Apoptotic cell death was detected by Hoechst staining. Nine cells per well/four wells per condition were plotted. **(A,C)** Data were analyzed using two-way ANOVA and Tukey’s HSD for pairwise comparisons. *p < 0.05. **(D)** RNAseq heatmap for expression levels of genes involved in the oxidative stress response for ABC, ARPE-19, and hRPE49 cells. **(E)** Evaluation of apoptosis and necrosis by flow cytometry relative to the increment in H_2_O_2_ concentration. Plots showing Annexin V vs. 7AAD signals were made (Supplementary Figure 4E) (100k events per condition). Four cell populations were analyzed in each experimental condition: 7-AAD+ cells for necrotic cells, 7AAD and Annexin V + cells for Late Apoptotic cells, Annexin V+ for Early apoptotic cells, and live cells negative for both 7AAD and Annexin V. Quadrants show the types of cell death detected differentiated by shape: Early and late apoptosis and necrosis.

**FIGURE 3:**
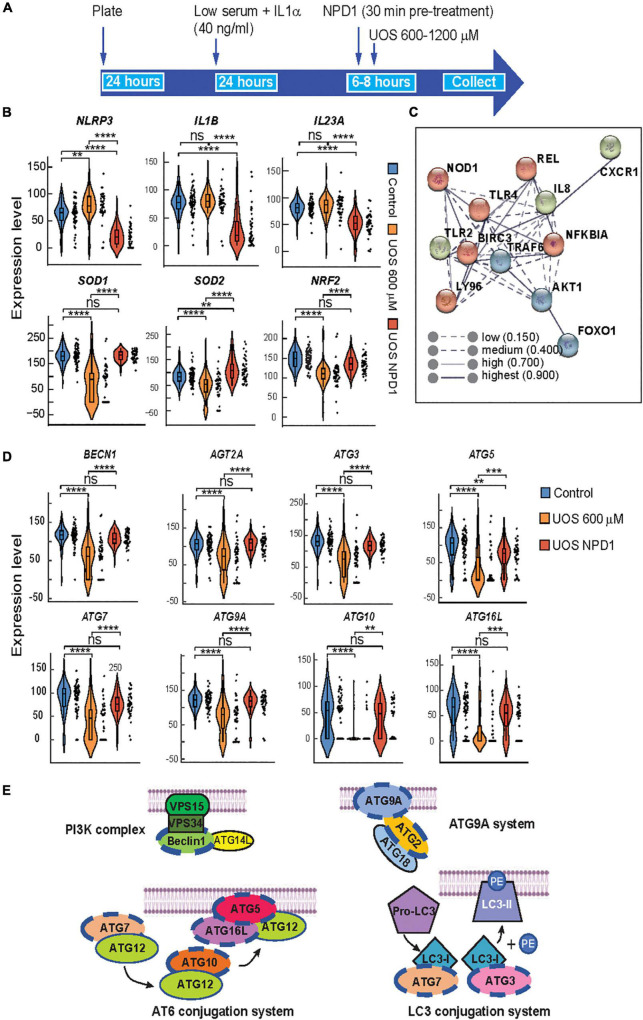
Autophagy and survival signaling are restored by NPD1 in ABC cells undergoing UOS. **(A)** Timeline of experiment for single-cell RT-PCR using Fluidigm Biomark. **(B)** Violin plots showing the expression of inflammasome-related genes. **(C)** STRING interaction network was generated for differentially expressed genes for NPD1-treated cells, showing high correlation and clustering of target genes involved in response to inflammation. **(D)** Violin plots showing autophagy-related genes. Y-axis illustrates the relative expression level for each gene. Each black dot at the right of the violin plots depicts a single-cell gene expression. One-way ANOVA was performed with the *Post Hoc* Tukey HSD test for multiple comparisons. *p < 0.05; **p < 0.01; ***p < 0.001; ****p < 0.0001. **(E)** Regulated genes tested by single-cell RT-PCR are involved in autophagy corresponding to the four depicted complexes that regulate the formation of the phagophore. Genes modulated by NPD1 are marked with a blue dashed line.

**FIGURE 4:**
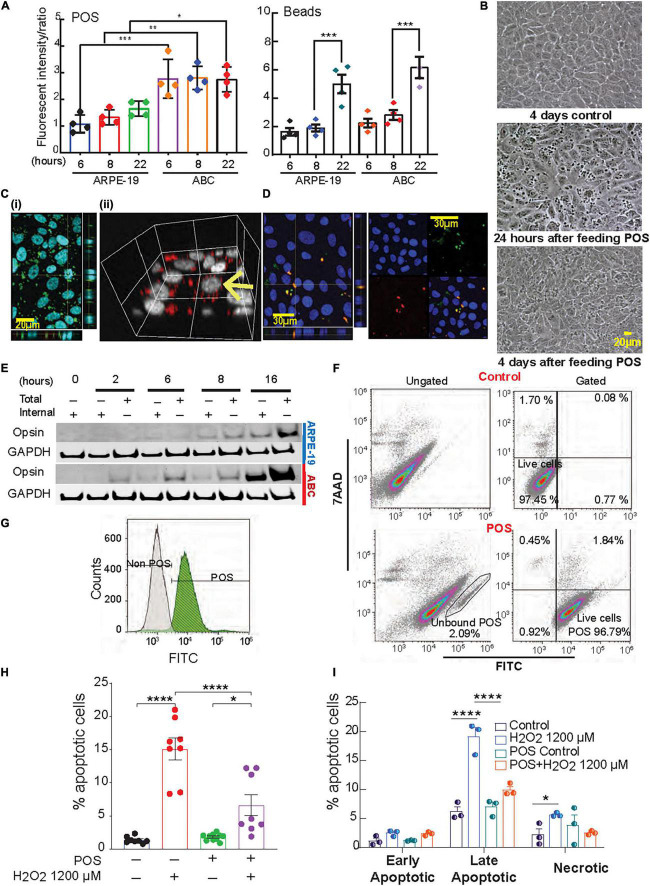
Phagocytosis activity in ABC is higher than ARPE-19 cells. **(A)** Phagocytosis of photoreceptor outer segments (POS) or polystyrene microspheres (beads) by ARPE-19 and ABC cells over time. Confocal microscopy was used to analyze bound and internalized FITC-labeled POS or fluorescent beads (Supplementary Figure 8). The ratio of relative fluorescence intensities was determined by measuring total fluorescence intensity of the green signal from the added material (beads or POS) over the total blue signal (Hoechst). **(B)** Live images showing ABC cells fed with and without POS. **(C)** Confocal images (z-stacks) of ABC cells show uptake of **(i,ii)** human POS and **(D)** rat photoreceptor outer segments; orthogonal view displays co-localization of the FITC labeled POS (green) and opsin (red). **(E)** Immunoblots with opsin antibody against the lysates of ABC and ARPE19 cells fed with bovine POS (Supplementary Figure 9 shows two independent biological whole membrane western blots). **(F,G)** Assessment of POS phagocytosis in ABC cells by flow cytometry. After gating out cell debris and doublets (Supplementary Figure 4A), and not bound/internalized POS **(F**, bottom left) in control or cells exposed to FITC-POS, 7AAD versus FITC signals were obtained (**F**, Gated, 100,000 events per condition were assessed). The great majority of viable cells (7AAD negative cell population) are POS + (bottom right). **(G)** Analysis of FITC signal alone showed a shift in the peak of it in ABC cells fed with FITC-labeled POS compared to one of the control ABC cells (white). **(H,I)** Quantitative analysis of Hoechst-stained ABC cells by fluorescence microscopy of POS phagocytosis effects on apoptotic cell population after oxidative stress. **(H)** Analysis of apoptosis by Hoechst staining. **(I)** Quatification of panel **(F)**. **(A,H,I)** One Way ANOVA and Tukey’s HSD for pairwise comparisons was applied. Results represent averages ± SEM of repeats of at least three independent experiments. *p < 0.05; **p < 0.01; ***p < 0.001; ****p < 0.0001.

### Assessment of Phagocytosis by Western Blot

ABC and ARPE-19 cells were incubated with unlabeled POS suspension for 0, 2, 6, 8, and 16 h. To stop the reaction, the cells were washed 3 times with PBS-CM buffer (PBS supplemented with 1 mM MgCl_2_, 0.2 mM CaCl_2_). To detect internalized POS only, the cells were washed 1 time with PBS, then incubated with PBS-EDTA for 5–10 min. Samples designated for total POS detection remained in PBS-CM. The PBS-EDTA was then removed, and the cells were washed three times with PBS-CM. The PBS-CM was removed from all wells, and the cells were lysed with RIPA buffer freshly supplemented with a protease inhibitor cocktail. Analysis of phagocytosed POS content in samples was determined by SDS-PAGE electrophoresis and opsin immunoblotting.

### Lipid Extraction and LC-MS/MS-Based Lipidomic Analysis

Cells were grown for 7–10 days before being collected to maximize phagocytosis and utilization and incorporation of lipids from outer segments into the RPE cells. The cells were then washed with ice-cold PBS with calcium to remove any unbound outer segments, and the lipids were extracted. Lipid extraction was performed similarly to our previous work (Do et al., 2019). Briefly, each sample was homogenized in MeOH (3 ml), followed by the addition of CHCl3 (6 ml) and the internal lipid standards (Cayman, Ann Arbor, MI, United States). After sonication in a water bath, samples were centrifuged, and the supernatant was added with pH 3.5 H_2_O for phase separation. The bottom phase (organic phase) was dried down under N2 and reconstituted in an AcN:MeOH:CHCl3 (90:5:5) solution. A Xevo TQ-S equipped with Acquity UPLC BEH HILIC 1.7 μm 2.1 × 100 mm column was used with solvent A (acetonitrile:water, 1:1; 10 mM ammonium acetate pH 8.3) and solvent B (acetonitrile:water, 95:5; 10 mM ammonium acetate pH 8.3) as the mobile phase. Solvent B (100%) ran for the first 5 min isocratically was graduated to 20% solvent A for 8 minutes, and then ran at 65% of A for 0.5 min. It ran isocratically at 65% of A for 3 min and then returned to 100% of B for 3.5 min for equilibration. The capillary voltage was 2.5 kV, the desolvation temperature was set at 550°C, the desolvation gas flow rate was 800 l/h, cone gas was 150 l/h, and nebulizer pressure was 7.0 Bars with the source temperature at 120°C.

### Matrix−Assisted Laser Desorption/ionization Imaging Mass Spectrometry

Matrix−assisted laser desorption (MALDI) was carried out as described previously (Kautzmann et al., 2020). Briefly, coverslips with cells were attached to MALDI plates and then placed within the sublimation chamber, where matrix (2,5-dihydroxybenzoic acid, DHB) was applied for positive ion mode analysis. Sections were then rasterized by laser, 355 nm, 2000 Hz Scanning control (15 μm, horizontal and vertical movement) and analyzed. Differential spectra represent relative abundance of lipid molecular species detected by matrix−assisted laser desorption/ionization imaging mass spectrometry (MALDI IMS) based on cell type were created.

### Isolation of Single Cells and cDNA Synthesis

Control stressed and treated single ABC cells were captured with the C1™ platform (Fluidigm Inc., South San Francisco, CA, United States) using Fluidigm’s Integrated Fluidic Circuits™ (IFC) according to the manufacturer’s instructions (Supplementary Figure 7A). Captured cells were imaged on IFC to confirm the number of cells per site, and the viability of the cell was confirmed using a LIVE/DEAD cell assay (Life Technologies, Waltham, MA, United States, Cat# L3224). Only single, viable cells were used for subsequent analysis. In the IFC, individual cells were lysed, and mRNAs were reverse transcribed and amplified to complementary DNAs. The resulting cDNAs from individual cells were collected from the IFC and diluted three times in TE buffer before utilization in the qPCR reaction with Biomark™.

### High Throughput qPCR by Biomark™

The qPCR reaction mixture had a volume of 5 μl and contained 2.25 μl of diluted preamplified cDNA, 0.25 μl of DNA Binding Dye (Fluidigm), and 2.5 μl SsoFast EvaGreen Supermix with low ROX (Bio-Rad, Hercules, CA, United States). The primer reaction mixture had a final volume of 5 μl and contained 2.5 μl Assay Loading Reagent (Fluidigm, San Francisco, CA, United States) and 0.25 μl of a mix of all reverse and forward primers (Supplementary Table 1), corresponding to a final concentration of 500 nM in the reaction. The Biomark 96.96 IFC was first primed with an oil solution in the Juno Controller (Fluidigm) to fill the fluidic circuit. Ninety-six sample reactions (5 μl each) were loaded into individual sample wells, and 96 forward and reverse primer mixtures were loaded into each assay well (5 μl each). The IFC was then placed in the Juno Controller for automatic loading and mixing. After an hour and a half, the IFC was then transferred to the Biomark™ HD qPCR platform (Fluidigm). The cycling program consisted of Thermal Mix at 70°C for 40 min followed by 60°C for 30 s. Hot Start was 1 min at 95°C, followed by 30 cycles of denaturation at 96°C for 5 s, annealing at 60°C for 20 s. Melting curves were collected between 60°C and 95°C with 1°C increments/3 s. We designed a set of 96 primer pairs (Supplementary Table 1) that comprise inflammatory and autophagy pathways in addition to housekeeping genes. The induction of UOS was optimized for the ABC cells by testing a range of H_2_O_2_ concentrations (Supplementary Figure 3). Naïve ABC cells or cells treated with H_2_O_2_ were isolated using the Fluidigm C1 microfluidics platform. Cells were captured into individual chambers, lysed, and their RNA reverse transcribed and amplified into cDNA. cDNA from single cells was collected, and qPCR was performed using the Fluidigm Biomark™ platform (Supplementary Figure 7A). Specific amplification of each targeted cDNA was confirmed by melt curve analysis. Measured Ct values were exported from the BioMark™ software to Excel for data analysis. Ct values of target genes were extracted through the Fluidigm Real-Time PCR Analysis program. The Ct value of target genes was normalized to the housekeeping genes.

### RNAseq Analysis

For RNAseq ABC cells, ARPE-19 cells, and hRPE49 cells were used. Cells seeded in 6-well plates (500,000 cells/well) were grown for at least 72 h. The total RNA was extracted using Trizol, and RNA quality and integrity were verified using NanodropOne (Thermo Fisher Scientific) and Agilent 2100 Bioanalyzer RNA nanochips. Library preparation was done using TrueSeq RNA library prep kit v2 – Set A (catalog #RS-122-2001) (Illumina); libraries were denatured, pooled, and normalized according to Illumina’s sample preparation guide. RNASeq was done on Illumina NextSeq 500 sequencing system using NextSeq 500 High Output v2 kit (150 cycles) (catalog# FC-404-2002).

### Bioinformatic Analyses of RNAseq and Single-Cell RT-PCR

The RNA reads were mapped to the human genome and processed through bioinformatics pipeline in QIAGEN CLC Genomics Workbench (Qiagen, Redwood City, CA, United States). Then the results were analyzed using R and heat map of gene expression was generated with Heatmap generator. Then the results are analyzed using Ingenuity Pathway Analysis tool (Qiagen, Redwood City, CA, United States). Principal component analysis (PCA), hierarchical clustering, violin plots, and box plots were carried out using the Bio Vinci program (Bioturing Inc., San Diego, CA, United States), GraphPad Software (La Jolla, CA, United States, United States)^2^ and Partek Genomics Suite software (Partek Inc., St. Louis, MO, United States).^3^ Gene Interaction Prediction was performed using STRING database^4^ (Szklarczyk et al., 2017).

### Statistics

Data were plotted using GraphPad Software, San Diego, CA, United States.^5^ Data are presented as means ± SEM. Comparisons were performed using ANOVA two-way. *P*-values were indicated as follows: ns *P* > 0.05, ^∗^*P* ≤ 0.05, ^∗∗^*P* < 0.01, ^∗∗∗^*P* ≤ 0.001, ^∗∗∗∗^*P* ≤ 0.0001. Data were filtered by *P* ≤ 0.05 and absolute value fold change ≥ 1.5.

### Illustration

The autophagy pathways illustration (Figure 6E) was adapted from Intartaglia et al. (2021) and created with BioRender.com.

**FIGURE 5:**
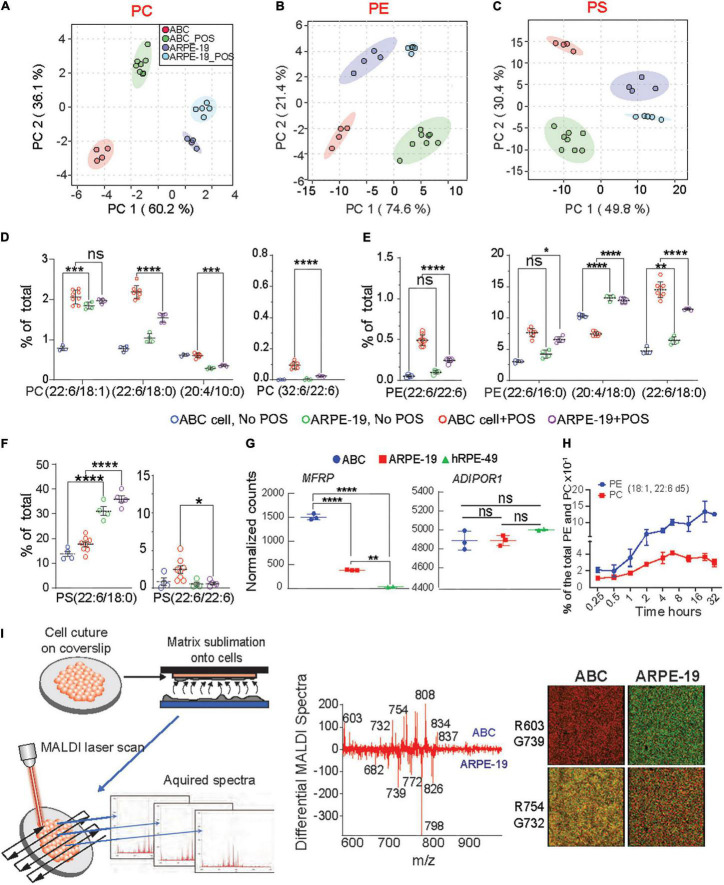
Enhanced incorporation and synthesis of phospholipids containing very-long-chain fatty acids in ABC cells. **(A–C)** Principle component analysis (PCA) were performed for PC, PE, and PS phospholipid species for ABC cells, and ARPE-19 cells after phagocytosis of POS. **(D–F)** LC-MS/MS quantitative distributions in bar graphs for ABC vs. ARPE-19 lipid species with and w/o POS feeding shows the most abundant PLs species found in the cells after feeding, containing either DHA and VLC-PUFAs (iv) or double DHA (iii). Tukey’s HSD *post hoc* testing was performed for two-factor ANOVA ****p < 0.0005, ***p < 0.005, **p < 0.05, *p < 0.01. **(G)** RNA sequencing analysis showing normalized counts for *MFRP* and *ADIPOR1* gene expression for each cell type. **(H)** ABC cells fed with deuterated DHA for up to 32 h were analyzed using LC-MS/MS. Line graphs showing two of the most abundant PL species (18:1, 22:6) containing DHA-d5 from ABC cell lysates, demonstrating the ability of the ABC cells to incorporate 22:6. **(I)** MALDI lipidomic comparative analysis. Coverslips with cells were attached to MALDI plates and then placed within the sublimation chamber, where matrix (2,5-dihydroxybenzoic acid, DHB) was applied for positive ion mode analysis. Sections were then rasterized by laser, 355 nm, 2000 Hz Scanning control (15 μm, horizontal and vertical movement) and analyzed. **(Middle panel)** Differential spectra show relative abundance of lipid molecular species detected by MALDI IMS based on the cell type. Molecules more abundant in ABC cells are presented in the upper part of the graph, while molecules more abundant in ARPE-19 are displayed at the bottom; m/z 834 corresponding to PC containing DHA (PC (18:0/22:6) was more abundant in ABC cells than in ARPE-19 cells. PC (16:0/18:1) with K + adduct (m/z 798) was the most dominant molecule in ARPE-19. **(Right panel)** Bicolor (red and green) image composition of lipid signals from MALDI IMS extracted from the cells and generated with positive ion mode. Same m/z shows different abundance from one cell type to another.

**FIGURE 6:**
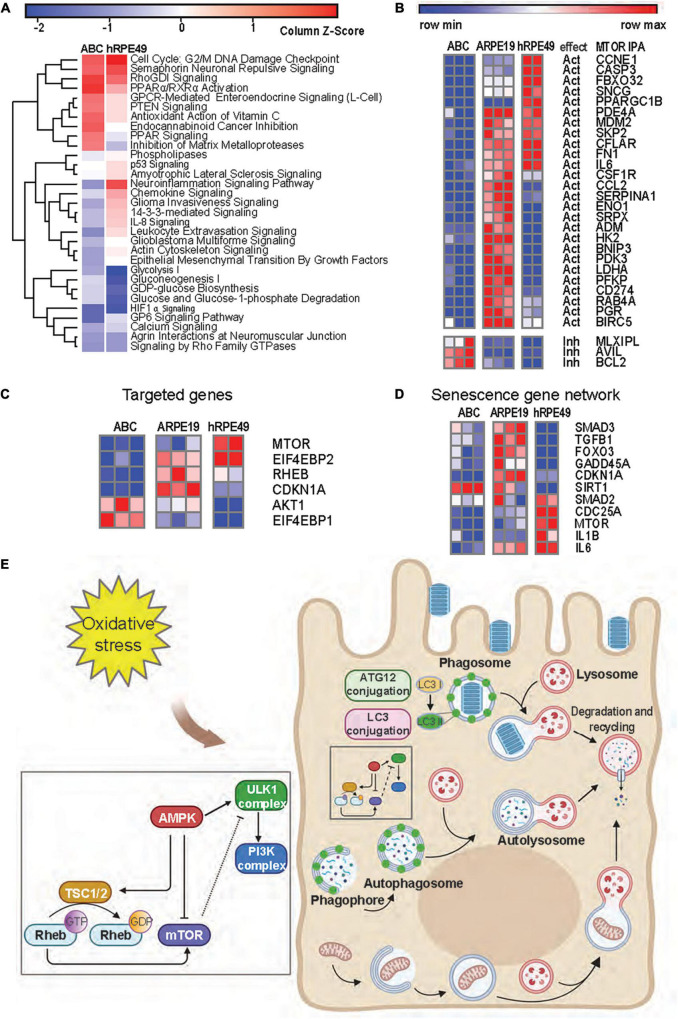
mTOR signaling is reduced in ABC cells as compared to ARPE-19 cells. **(A)** Heatmap from RNA seq analysis of ARPE-19 cells compared to ABC and hRPE49 cells showing canonical pathways with the most significant changes. IPA analysis was performed using ARPE-19 cells as control. Red color means the pathway is upregulated when compared with ARPE19, while blue means it is downregulated. **(B)** Comparative heatmap representation of RNA seq data of mTOR downstream signaling genes (Supplementary Figure 10 shows IPA network). The expected effect of mTOR on its downstream targets is labeled as “Act” for activation and “Inh” for inhibition. **(C)** Analysis of genes involved in geroconversion and autophagy activation by mTOR signaling and its related genes. **(D)** Heatmap analysis of genes involved in senescence. **(E)** Autophagy pathways in RPE cells.

## Results

### Human Primary Cells That Phenotypically Resemble RPE in the Human Eye

ABC cells were obtained from retina xenografts of a 19-year-old Caucasian male (Figure 1A). At passage 8 and over, they conserved the pigmentation (Figure 1A, right panel) and extensive areas of polygonal-shaped cells at higher densities (Figures 1E,F). We compared the transcription profile of ABC cells, ARPE-19, and a primary culture originating from a 49-year-old Caucasian male using the same technique utilized for ABC by means of RNAseq. The latter, cultured hRPE49, disclosed a limited life span, reaching passage 9 at most. ABC and ARPE-19 both originated from 19-year-old Caucasian males; the second underwent spontaneous immortalization (Dunn et al., 1996). Of the three cell lines compared, ABC depicted the most abundance of RPE markers. Retinal dehydrogenase 5 (RDH5); Bestrophin 1 (BEST1); MER proto-oncogene, tyrosine kinase (MERTK); retinaldehyde binding protein 1 (RLBP1); Paired box gene 6 (PAX6); Orthodenticle homeobox 2 (OTX2); Microphthalmia-Associated Transcription Factor (MITF); retinoid isomerohydrolase RPE65 (RPE65); cellular retinaldehyde-binding protein (CRALBP); and pigment epithelium-derived factor/serpin family F member 1 (PEDF/SERPINF1) are essential for the function and maintenance of RPE cells and photoreceptors, and their mutation causes retinal degenerating pathologies (Housset et al., 2013; Raviv et al., 2014; Xue et al., 2015; Liu et al., 2017; Audo et al., 2018; Guziewicz et al., 2018; Chen et al., 2019; Ma et al., 2019; Wang et al., 2020; Zhang et al., 2020). These genes are expressed in ABC, and some are underrepresented in the other two cell lines (Figure 1B, right panel). Thus, ABC represent a good *in vitro* model to study the role of these genes and their associated retinal diseases. Single-cell Real-Time PCR showed uniform expression of cellular retinaldehyde binding protein (*CRALBP*), *RPE65*, and *OCCLUDIN*, a tight junction protein. *RDH5*, which was represented more in hRPE49 by RNAseq (Figure 1B, right panel), evidenced more variability within the ABC cell population, with up to 50% of them exhibiting low expression (Figure 1C). RPE65, BEST1, RDH5, RLBP1, and SERPINF1 were confirmed using reverse transcription polymerase chain reaction (RT-PCR) and Real-Time polymerase chain reaction (qPCR) (Supplementary Figures 1A,B).

Retinal pigment epithelial cells derived from human embryonic stem cells (hESCs) have recently arisen as a model for RPE-related diseases. Nevertheless, hESC-derived RPE undergoes mesenchymal-epithelial transition in culture before acquiring an epithelial phenotype (Choudhary et al., 2015). ABC cells show minimal expression of mesenchymal cell markers as well as markers of mesenchymal-epithelial transition, thus displaying more phenotypic stability (Figure 1B, middle panels). Western blot analysis comparing ARPE-19, ABC, and fibroblasts illustrates that proteins Bestrophin-1 (BEST1) and Cytokeratin 8 (Figure 1D and Supplementary Figure 2) were only present in the two RPE cell lines, and BEST1 was the highest expressed in ABC, confirming the phenotype stability observed by the low expression of the mesenchymal and the MET transition (Figure 1B).

RPE cells generate three types of pigment melanin and fuscin. Melanosomes are developed during brief periods at embryonic stages, while fuscin accumulates with aging as lipofuscin and melanolipofuscin granules (Boulton, 2014). Two mRNAs coding for enzymes implicated in the synthesis of eumelanin from tyrosin, tyrosinase (TYR) and tyrosinase-related protein 1 (TYRP1), were found in high abundance in ABC in comparison to ARPE-19 and hRPE-49. Premlanosome protein mRNA, PMEL, was also present, although in less abundance (Figure 1B, left panel). These data confirm the ability of ABC to produce melanin-like granules *in vitro* (Figure 1A).

Unlike other human RPE cell lines, ABC were capable of retaining their original characteristics along passages (Figures 1A,E). The expression of GFP-tagged-membrane frizzled-related protein (GFP-MFRP), which is localized in cytoplasm and membrane, exhibited filopodial processes (Figure 1F). After two weeks in culture, these cells depicted a classical RPE hexagonal shape (Figure 1F). Moreover, villar processes appeared on the apical surface when they formed a packed monolayer with well-defined tight junctions (Figure 1G, middle panels). ABC cells pelleted, embedded in plastic, and thin-sliced displayed prominent apical villar processes and a single large central nucleus (Figure 1G, right panel). The formation of tight junctions was tested using a basal flux assay (Figure 1H, left panel) in which trypan blue was placed in the insert chamber, and it was allowed to diffuse freely toward the well through the permeable membrane. ABC cell tight junctions prevented the dye from diffusing across the monolayer, even after 12 h of incubation (Figure 1H, right panel and Supplementary Figure 4B). Immunostaining with Zona Occludens confirmed the formation of the tight junctions after one week of incubation (Figure 1I). Notably, the monolayers depicted incomplete cariocinesis, represented by cells containing more than one nuclei (Figure 1I, yellow arrow).

Additionally, microphthalmia-associated transcription factor (MITF), β-Catenin, and Peropsin displayed nuclear, peripheral, and cytoplasmic localization in ABC cultures accordingly with the distribution of the proteins in the monolayer (Figure 1J).

These results demonstrate that ABC cells display phenotypic and genetic features closer to the RPE native pigment epithelium than the other two cell lines regarding stability, purity, and structure, indicating that key proteins are correctly localized and that the expression of desirable enzymes for pigmentation and function occurs. From this point forward, we will present evidence to consider ABC a suitable model to use in AMD studies.

### Postmitotic ABC Cells Display UOS Resistance

To determine the proliferation rate variation in response to confluency and stress, a time curse was performed, and the dividing cells were detected using Ki67 staining in the presence or absence of UOS (Figures 2A,B). In parallel, apoptosis was measured using Hoechst staining. In naïve cells, Ki67 values declined from 100% at day 1 to 30% at day 8, reaching the 50% mark between day 5 and 6 (Figure 2A, red curve). When H_2_O_2_ was applied to induce uncompensated oxidative stress, less than 1% of cells were Ki67 positive at day 1, and the number increased with time as the population recovered, reaching stabilization at around 50% between days 5 and 6 (Figure 2A, green curve). Days 1 and 2 depicted the higher values of apoptosis (Figure 2A, blue bars), whereas the sensitivity decreased drastically to 25% after 3 days of growth to become almost null by day 8. Accordingly, the appearance of the initial hexagonal-shaped cells occurs one week after plating at this density. Concentration curves confirmed these observations (Supplementary Figures 3A–D). Functional tight junctions appeared after 7 days of culturing ABC cells in inserts (Figure 1H). When ARPE-19 and ABC cells response was compared under the same conditions, the first cell line reached 60–65% cell death at 600 μM H_2_O_2_, but the second one did not yield significant differences from their own control (concentration 0 μM). ARPE-19 cell death was raised to 100% with 800 μM, while <75% of ABC cells died at 1200 μM (Figure 2C). In addition, the UOS response genes differ between ABC and ARPE19 cells, with 8 out of 14 genes highly expressed in ABC cells (Figure 2D), suggesting that in ABC cells, the antioxidant systems are set forth to promote protection against ROS. Notably, mRNAs coding glutathione peroxidases 2, 3, and 8 (GXP2, GXP3, and GXP8) were elevated in ABC. GXP2, GXP3, and GXP8 are three isoenzymes belonging to the glutathione peroxidase family that catalyze the reduction of organic hydroperoxides and hydrogen peroxide (H_2_O_2_) by glutathione, protecting the cells against oxidative damage (Nirgude and Choudhary, 2021; Ren et al., 2022; Yin et al., 2022). The mRNA encoding Thioredoxin reductase 1 (TXNRD1), a selenocysteine-containing enzyme involved in redox homeostasis (Snider et al., 2013), was over-represented in ABC as well. To determine whether necrosis and apoptosis were occurring, flow cytometry was used to analyze a population of cells stained with Annexin V and 7AAD. Dot charts and histograms indicate the presence of four populations of cells (Figure 2E and Supplementary Figure 4D). Four cell populations were analyzed: (1) live cells negative for both Annexin V and 7AAD, (2) early apoptotic cells positive for Annexin V, (3) cells positive for both Annexin V and 7AAD, demonstrating late-stage apoptosis, and (4) necrotic cells positive only for 7AAD (red population). A clear concentration-dependent decrease in the ABC viable cell population, with a concomitant increment in the late apoptotic population, is evident (Figure 2E). Overall, the majority of dying cells are apoptotic; only a small fraction (10%) are necrotic.

These results suggest ABC cells become postmitotic and increase resistance to uncompensated oxidative stress, probably through the activation of key glutathione-related homeostatic effectors that counteract the oxidized reactive species and stabilize the redox status of the cell.

### ABC Cells Undergoing UOS Are Rescued by NPD1

Neuroprotectin D1 synthesis is triggered by stress to promote homeostasis and survival in RPE cells (Calandria et al., 2009). NPD1 modulates the expression of proteins like BIRC3 (Calandria et al., 2009) to rescue RPE cells from apoptosis induced by UOS. To assess the transcriptional response of ABC to NPD1, single-cell RT-PCR was used (Figure 3A) to screen the expression of genes related to inflammasome activation and autophagy, both of which have been widely implicated in the pathogenesis of AMD (Mitter et al., 2014; Celkova et al., 2015). Two different concentrations of H_2_O_2_ modulate the expression differentially (Supplementary Figures 5, 6). NPD1 uniformly downregulated the expression of NLRP3 (3-fold), IL1B (4.2-fold), and IL23 (1.8-fold) when compared to cells undergoing UOS. Conversely, NPD1 augmented the expression of antioxidant and anti-inflammatory genes, SOD1 (2.1-fold), SOD2 (1.6-fold), and NRF2 (1.5-fold) in comparison to the UOS-only treated cells (Figure 3B). Then, RELB, cREL, and BIRC3 expressions were compared to define the mechanism of action of NPD1 in ABC cells (Supplementary Figure 7B). The distribution of cells expressing each gene exhibited bimodal expression patterns. NPD1 triggered the expression of RELB in a large population of cells compared to UOS alone or control. NPD1 brought up cREL expression to control levels in cells undergoing UOS and upregulated BIRC3 expression. Not all cells responded to the stress or NPD1, denoting a high heterogeneity in the response. The monophasic distribution of TFRC expression across the three conditions attested to the preservation of the transcriptional machinery in all the cells analyzed (Supplementary Figure 7B). To identify functional connections between the relevant genes, a protein-protein interaction network was constructed using the STRING database (Figure 3C; Szklarczyk et al., 2017). We used the top differentially expressed genes with *P*-values < 0.01 as inputs for the STRING database to determine the molecular network of interacting genes and to obtain correlations with a high probability confidence score (≥0.900). The protein-protein interaction analysis performed using STRING indicated that the proteins differentially represented in ABC cells are highly correlated (*P*-value < 1.04e-10). The results were significantly enriched with BIRC3 and TLR4 as the main nodes in the network.

Autophagy plays an important role in maintaining cellular homeostasis by eliminating damaged organelles and protein aggregates and by removing infectious agents from host cells (Cho and Hwang, 2012; Szatmári-Tóth et al., 2016; Harris et al., 2017). Defects in autophagic machinery generate sensitivity to oxidative stress conditions in RPE cells (Mitter et al., 2014), and it is associated with several disorders, including AMD (Kaarniranta et al., 2017) and neurodegenerative and infectious diseases (Jing and Lim, 2012). Furthermore, the interest in the components of this pathway is enhanced by the fact that RPE cells utilize part of the autophagy complexes to perform phagocytosis of the photoreceptor tip renewal (Mitter et al., 2014). To determine whether NPD1 affects autophagy, ABC cells undergoing UOS were treated with NPD1, and a panel of autophagy-related genes was evaluated using single-cell Real-Time PCR. NPD1 consistently restored the expression of autophagy-related genes to homeostatic levels in single ABC cells (Figure 3D). Beclin1 (BECN1), autophagy-related proteins 9A and 2 (ATG9A and ATG2A), which are regulatory parts of the PI3K complex and ATG9A system involved in the nucleation of the Phagophore, were downregulated in a subpopulation of cells by UOS and restored by NPD1 (Figures 3D,E, blue dash line). Similarly, ATG7 and ATG3, which are part of the LC3-conjugation system, are downregulated by UOS in a subpopulation and totally recovered by NPD1. Intriguingly, ATG10, ATG5, and ATG16L, which are contained in the ATG12-conjugation system, are downregulated by UOS in the majority of the cells since this complex is the main candidate for disruption by UOS and the levels are brought back to control levels but not in the totality of the cells. These two last complexes work on the conjugation of LC3 to the lipid phosphatidylethanolamine (PE) in the elongation step of the phagophore and the addition of LC3 to the endosome in the LC3 associate phagocytosis.

Therefore, NPD1 contributes to the resistance of ABC cells against UOS by modulating genes involved in inflammasome, apoptosis, antioxidant pathways, and autophagy.

### Active Biosynthesis of VLC Fatty Acid Species Indicates an Outer Segment Cycle Renewal Mechanism Preservation Is Expressed in ABC Cells

During daily cycle renewal, the outer segments of the photoreceptors are phagocytized by the mammalian RPE cells, which in turn recycle visual pigment components and DHA in a circadian manner (Bok, 1993; Kolko et al., 2007; Bazan et al., 2010; Asatryan and Bazan, 2017). In fact, RPE cells are among the most phagocytic cells in the whole organism (Mazzoni et al., 2014). To evaluate phagocytosis, ABC and ARPE-19 cells were incubated with Fluorescein isothiocyanate (FITC)-labeled bovine POS or fluorescent polystyrene beads. Both cell types actively ingested these particles with relative fluorescence abundance higher in ABC cells compared to ARPE-19, especially for POS (Figures 4A,B and Supplementary Figure 8), suggesting that the phagocytic activity was greater in ABC cells. Confocal imaging, along with 3D rendering of human, rat, and bovine POS fed to confluent ABC cells, revealed their intracellular localization (Figures 4C,D). Additionally, opsin immunoblotting signal for both internal and total opsin on immunoblots increased with time, reaching its maximum at 16 h (Figure 4E and Supplementary Figure 9). There was a 4-fold increase in opsin internalization in ABC cells compared to ARPE-19 cells (Figures 4A,E). To further confirm the findings, the uptake of labeled bovine POS was analyzed using flow cytometry and 7AAD counterstaining to detect viable cells. The majority of viable cells were POS-positive (Figure 4F, bottom right, Figure 4G). The gating for Hoechst signal to separate debris from cells and for FITC signal to detect endocytosed labeled POS peak shifted when ABC cells fed with POS were compared to control (Figure 4G), implying that the majority of FITC-POS have been phagocytized and internalized.

Phagocytosis triggers the biosynthesis of protective lipid messengers that have an autocrine effect on RPE cells (Mukherjee et al., 2007). In ABC cells exposed to 1200 μM H_2_O_2_, the phagocytosis of POS attenuated UOS-induced apoptosis (Figure 4H). Flow cytometry was used to determine the populations of early, late apoptosis, and necrotic cells for the different treatments. Compared to control only, cells incubated with POS under UOS conditions exhibited a negligible amount of necrosis and early apoptosis. Late apoptosis was the predominant form, and the population was significantly smaller than the cells undergoing UOS alone (Figure 4I).

ABC and ARPE-19 cells were then fed with bovine POS at a 1:10 ratio (cell:POS), and their membrane lipidome was screened using LC-MS/MS and MALDI. Principal component analysis (PCA) indicate that phosphatidylcholine (PC), phosphatidylethanolamine (PE), and phosphatidylserine (PS) species form distinctive clusters differentiating cell type and presence or absence of POS (Figures 5A–C). Random forest PL analysis confirmed differences (Supplementary Figure 10A). Moreover, phospholipids with DHA and VLC-PUFAs, lipids that are not considered integral to their membranes, were found in ABC cells after phagocytizing POS (Figures 5D–F). ARPE-19 cells fed with POS increased PL species containing DHA and VLC-PUFAs to a lesser extent than the ABC cells (Figures 5D–F). The main two phospholipids in the eyes and brain are phosphatidylcholine and phosphatidylethanolamine. Some species in all three types of PL were found to contain docosahexaenoic acid (DHA) of 22 carbons and 6 double bonds (22:6) and arachidonic acid (AA) of 20 carbons and 4 double bonds and other fatty acids omega 6 backbones (Figures 5D,F). Species of PE containing AA were more abundant in ARPE-19 despite whether they had undergone phagocytosis of POS or not (Figure 5E). PC species contain a fatty acid of 32 carbons and 6 double bonds (32:6), a precursor of Elovanoid 32, in addition to DHA (Figure 5D). PE and PS both depict species with two DHA molecules, one in sn1 and the other in sn2 (Figures 5E,F). In all the cases except PC(18:0; 22:6) and PE(20:0; 22:6), species containing DHA were increased in ABC cells above the levels observed on ARPE-19 after being fed POS. AdipoR1 and MFRP, contribute to the conservation of DHA in the retina (Rice et al., 2015; Kautzmann et al., 2020). The two genes are expressed in ABC, ARPE-19, and hRPE49 cells, with a higher basal expression of AdipoR1 in ABC cells (Figure 5G). ABC cells were then incubated for 32 h in the presence of deuterated DHA to determine the incorporation of the fatty acid into the phospholipids (Figure 5H). PE species showed an increase in deuterated DHA content steeper than PCs when species containing 18:1 and 22:6 were measured as representative phospholipids (Figure 5H). To compare the two cell types imaged by MALDI IMS, a difference spectrum was constructed, subtracting the ARPE-19 cell profile from the ABC cell profile (Figure 5I). The resulting relative plots emphasized lipid abundance, with the prevalent ABC cell lipids (pointing up) and the ARPE-19 lipids as downward peaks. DHA−containing PC [m/z 834, PC (18:0/22:6)] was more prevalent in the ABC cells compared to ARPE-19 cells, whereas PC [m/z 798, PC(16:0/18:1)K^+^ and m/z 782 PC(16:0/18:1)Na^+^] was enhanced in the other cell types. Image extraction of specific m/z (m/z 603, 739, 754, and 732) from the whole lipid spectrum allowed us to visualize the relative intensity difference between the ABC and ARPE-19 cells (Figure 5I, right panel). Overall, this comparison suggests that the ABC cells have a lipid-membrane composition enriched in DHA-containing PLs.

### Inhibition of mTOR Explains Why ABC Cells Do Not Become Senescent, a Key Event in AMD

Senescence plays a major role in tumor suppression, aging, tissue repair, and embryonic development (Cho and Hwang, 2012; Leontieva et al., 2014; Sun, 2014). Senescence occurs in two steps of cell arrest, which is common to quiescence, and geroconversion, which makes senescence irreversible (Blagosklonny, 2014). Contact inhibition is a form of quiescence where cell growth is arrested by contact, and it can be reversed when cells are passaged at low density. Pathways associated with geroconversion are p53 and mTOR (Blagosklonny, 2018), while consequences of the decreased senescent program can be noted by the downregulation of IL-8 signaling and PTEN signaling (Jung et al., 2019; Figure 6A). Suppression of mTOR is associated with contact inhibition in normal cells, including RPE cells (Cho and Hwang, 2012; Leontieva et al., 2014). mTOR pathway activation favors senescence gene programming and expression of the senescence-associated secretory phenotype (SASP), consisting of inflammatory cytokines (IL-1β, IL-6, or IL-8), growth factors, and proteases (Di Micco et al., 2021). The comparison between the expression signatures of ABC vs. ARPE-19 and hRPE49 vs. ARPE-19 using IPA depicted a reduced neuroinflammatory signaling pathway for the ABC cells (Figure 6A). Moreover, decreased glycolysis, gluconeogenesis, and lipolysis suggest that fully confluent ABC cells stop dividing and require less energy to sustain survival than ARPE-19 cells, which do not stop growing and dividing even after becoming confluent, therefore, requiring substantial energy (Figure 6A). Except for three of the genes plotted, the remaining mRNAs belonging to the mTOR pathway were downregulated in ABC cells in comparison to the other two cell lines inhibited in our data set, which coincides with the IPA prediction (Figures 6B,C; Supplementary Figure 10B). Low energy in the cell results in phosphorylation and activation of the TSC2/TSC1 complex formation, yielding inactivation of RHEB and, thus, inactivation of mTOR (Figures 6C,E). These results suggest that ABC cells develop a state of contact inhibition but not senescence when they become fully confluent. The senescence SASP network, like IL-1β and IL-6, are downregulated in ABC, and SIRT1 is upregulated (Xu et al., 2020), which marks the note that senescence is reduced in ABC cells (Figure 6D and Supplementary Figure 10B). In addition, mTOR signaling acts as an initiator of autophagy, and it is involved in the LC3-conjugation for the maturation of the phagosome in the LC3-associated phagocytosis (Figures 6E,4E; Intartaglia et al., 2021). Therefore, ABC cells display phenotypical, genetic, and functional characteristics that resemble the native pigment epithelium of the retina, in which the processes and signaling pathways convey the main cellular tasks that define the normal dynamics of the tissue.

## Discussion

We have developed a human RPE cell that recapitulates native features of those cells in the human eye. RPE cells are essential for photoreceptor functions and integrity, and our cell line provides opportunity to discern the fundamental properties and potential of this cell.

ABC cells become quiescent at 100% confluency due to contact inhibition (Figure 2A), and due to a decreased mTOR pathway activity, they resume growing if replating at lower densities. Two opposing events arise in primary RPE (hRPE49) and ARPE-19 cells. The former stop dividing, become senescent, and die when they reach 100% confluency, while ARPE-19 cells sustain viability for many weeks, even at post-confluency. The mechanisms behind this difference can be explained on the basis of mTOR signaling. Cellular senescence is triggered by many stimuli, including telomere attrition and DNA damage, oxidative stress, oncogene activation, and deactivation of tumor suppressor genes. Senescence plays a major role in tumor suppression, aging, tissue repair, and embryonic development (Cho and Hwang, 2012; Leontieva et al., 2014; Sun, 2014). The key feature of senescence is irreversibility, in contrast with quiescence from growth factor deprivation, which can be reversed. Contact inhibition is a form of quiescence where cell growth is arrested due to cells in contact with each other once high density is achieved. Then cells resume division when passaged and cultured at low density. The mTOR pathway is critical for geroconversion, the conversion of reversible cell cycle arrest to senescence (Leontieva et al., 2014). Suppression of mTOR is associated with contact inhibition in normal cells, including RPE cells (Cho and Hwang, 2012; Leontieva et al., 2014). mTOR pathway activation favors senescence gene programming and expression of the senescence-associated secretory phenotype (SASP), consisting of inflammatory cytokines (IL-1β, IL-6, or IL-8), growth factors, and proteases (Di Micco et al., 2021). The canonical pathways for the ARPE-19 transcriptomic dataset obtained by RNAseq were compared to ABC and hRPE49 datasets, and reduced glycolysis, gluconeogenesis, and lipolysis were found, suggesting that the fully confluent ABC cells stop division, requiring less energy to survive than ARPE-19 cells. On the other hand, ARPE-19, which do not stop growing even after becoming confluent, require substantial energy to thrive (Figure 6A). Consistent with this observation, the mTOR pathway is downregulated in ABC cells compared to ARPE-19 or hRPE49 cells (Figures 6B–D), while the Tuberous Sclerosis Complex 2 (TSC2) pathway is activated (Figure 6C). Twenty-six out of 29 genes in the mTOR pathway were inhibited (Figures 6B,C). Low energy in the cell results in phosphorylation and activation of the TSC2/TSC1 complex formation, yielding inactivation of RHEB and, thus, inactivation of mTOR (Figure 6E). This pathway is central for cellular growth and promotes translation by acting on eukaryotic initiation factor 4E (elF-4E), which needs to form complexes to activate the translation of cap-mRNA. Once 4E is associated with BP1, it is inhibited. Intriguingly, the mTOR activation is intrinsically involved in the initiation of LC3-conjugation leading to autophagy and LC3-associated phagocytosis (Figures 4E, 6E) related to the phagocytosis of the POS, and signaling here comes full circle (Intartaglia et al., 2021). mTOR signaling activation/deactivation equilibrium is of importance in the survival of the retinal pigment epithelium (Zhang et al., 2021) and the reduced gene expression of their component in ABC cells allows them to upregulate the mechanism on demand when the cell needs to, for instance, activate phagocytosis, a process that is not perturbed in this cell line (Figure 4).

The ability of ABC cells to form monolayers resembling the native retinal pigment epithelium is demonstrated by their formation of microvilli (Figures 1F,G), functional tight junctions that stop the passive diffusion of trypan blue (Figure 1H), and honeycomb packing culture (Figures 1A,E,I). RNAseq comparing ARPE-19 and ABC, both cell lines derived from 19-years-old Caucasian male donors, evidences significant differences in several important pathways related to senescence, inflammation, oxidative stress response, as well as markers of native RPE, which were more represented in ABC than in the first mention cell line. In regard to the oxidative stress response, ARPE-19 are more susceptible to cell death upon exposure to lower concentrations of H_2_O_2_ than ABC. The commercially available lowest passage for ARPE-19 is P19. At this passage, ABC undergo contact inhibition without stepping into senescence. ARPE-19, which were spontaneously immortalized (Dunn et al., 1998), displayed a reduced MET transition and Mesenchimal signaling that evidenced a stable RPE phenotype (Figure 1B). iRPE cells are a new development to model genetic diseases, such as L-ORD (Miyagishima et al., 2021), as well as expensive, time-consuming, and require expertise. Although this cell line offers several benefits, there are also certain limitations such as gender bias. The male origin of the cells may predispose the culture to enhance sensitivities, especially to steroids like testosterone (Penaloza et al., 2009). Our group is currently working to obtain a mirror culture originating from female donors. During embryonary stages, RPE cells originate from neuroectoderm, following common steps in the differentiation until reaching its final form, an epithelial-type cell. For this reason, RPE cells share many features with astrocytes and neurons (Bharti et al., 2011). This similarity was used to convert RPE cells into photoreceptors, a specialized type of neuron (Liang et al., 2006). Currently, exploration of this alternative from iPS cells is underway (Shrestha et al., 2020), and transdifferentiation of RPE cells, such as our ABC cells, into photoreceptors may offer a simpler option for cellular replacement therapies.

## Data Availability Statement

The original contributions presented in this study are included in the article/Supplementary Material, further inquiries can be directed to the corresponding author/s.

## Author Contributions

NB conceived the study. NB, AA, and JC designed the experiments and wrote the manuscript. JC generated the hRPE49 cell line. JC and AA analyzed the data. AA conducted the IHC experiments. M-AK conducted the beads and POS phagocytosis experiments and MALDI analysis. M-AK, JH, and AA performed the single-cell experiments. AA and BJ conducted the LC−MS/MS experiments. AA, BJ, and TP analyzed the resulting data. WG executed the bright field histology. KD performed the Ki67 analysis, the protein and gene expression phenotyping of the cells, and protein analysis of the phagocytosis experiment. VB and AA conducted the experiments for the POS phagocytosis and challenge with UOS. MVM analyzed the resulting data. SB and TP made the RNAseq gene analysis. JC, WG, M-AK, and NB edited the manuscript with input from all other authors. All authors have read and approved the manuscript.

## Conflict of Interest

The authors declare that the research was conducted in the absence of any commercial or financial relationships that could be construed as a potential conflict of interest.

## Publisher’s Note

All claims expressed in this article are solely those of the authors and do not necessarily represent those of their affiliated organizations, or those of the publisher, the editors and the reviewers. Any product that may be evaluated in this article, or claim that may be made by its manufacturer, is not guaranteed or endorsed by the publisher.

## Footnotes

1
http://imagej.nih.gov/ij

2
www.graphpad.com

3
https://www.partek.com/partek-genomics-suite/

4
http://string-db.org/

5
www.graphpad.com
