# Supplementary material for: New Retinal Pigment Epithelial Cell Model to Unravel Neuroprotection Sensors of Neurodegeneration in Retinal Disease
Source: Front Neurosci. 2022 Jun 30;16:926629. doi: 10.3389/fnins.2022.926629 (PMC9301569; doi:10.3389/fnins.2022.926629)
Supplement: Supplementary file 1 [file Data_Sheet_1.DOCX]

Supplementary Material

# Supplementary Figures

**
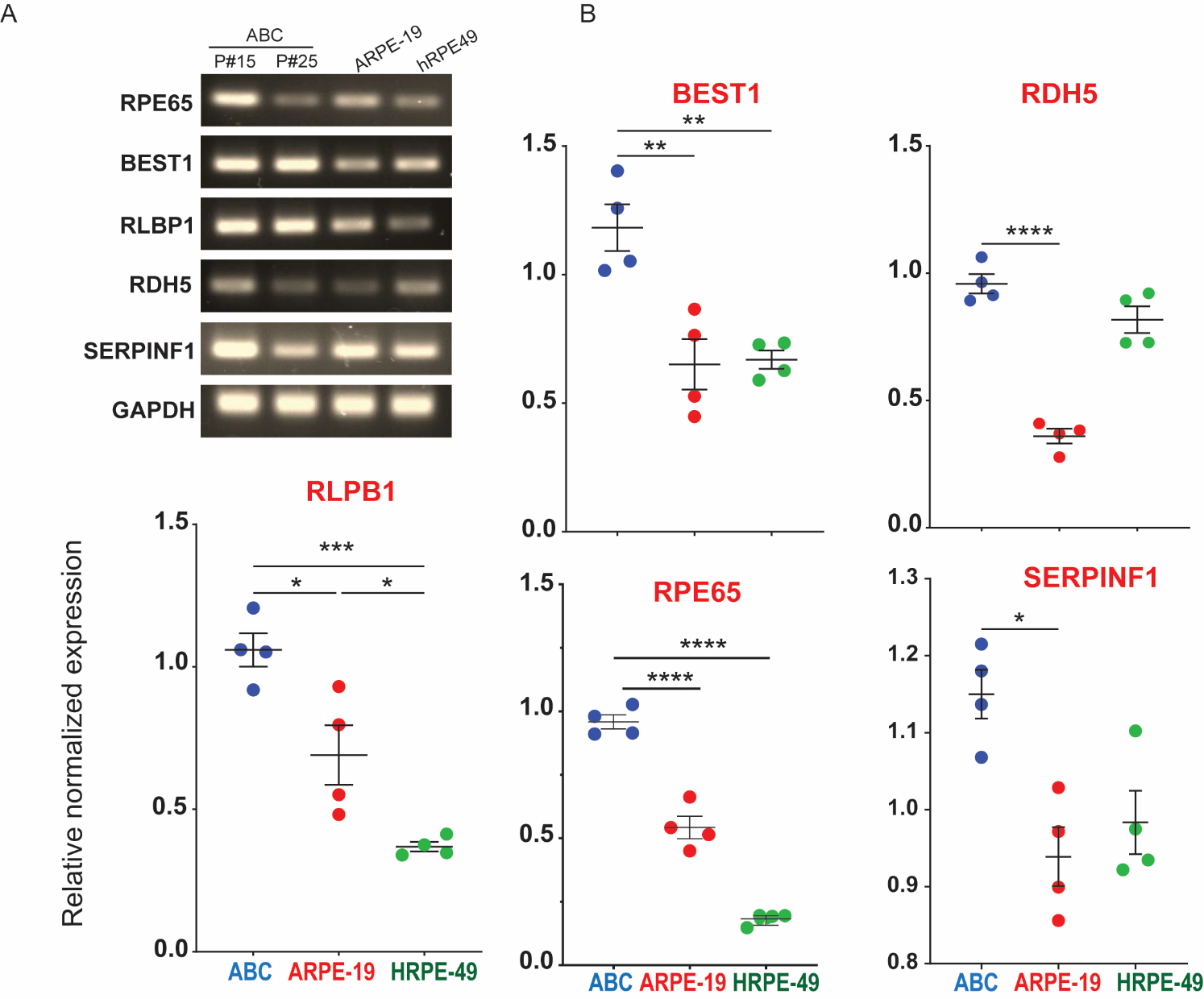
**

**Supplementary Figure 1.** **(A)** Quantitative PCR analysis showing the expression levels of RPE specific markers in early (P#15) and late (>P#25) ABC cells, ARPE-19, and in-house hRPE49 cells. **(B)** Comparative transcript expression analysis of RPE-specific genes evaluated by Real-time RT-PCR in ABC, ARPE-19, and hRPE49 cell lines.

**
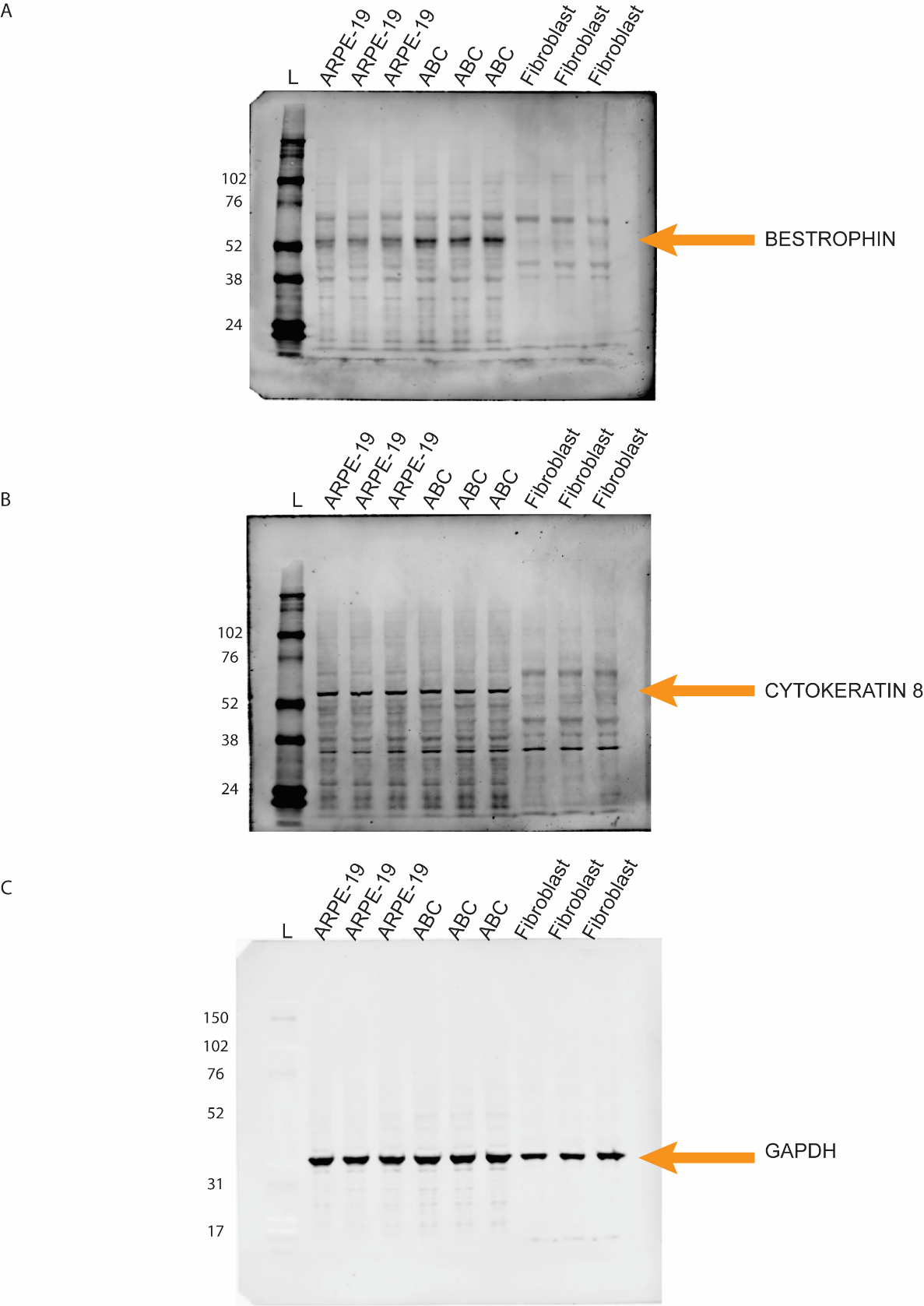
**

**Supplementary Figure 2.** Comparative protein expression levels of Bestrophin-1 (BEST1) and Cytokeratin-8 in ABC, ARPE-19, and fibroblast. GAPDH used as loading control. L, protein ladder. Molecular weights are in kDa.

**
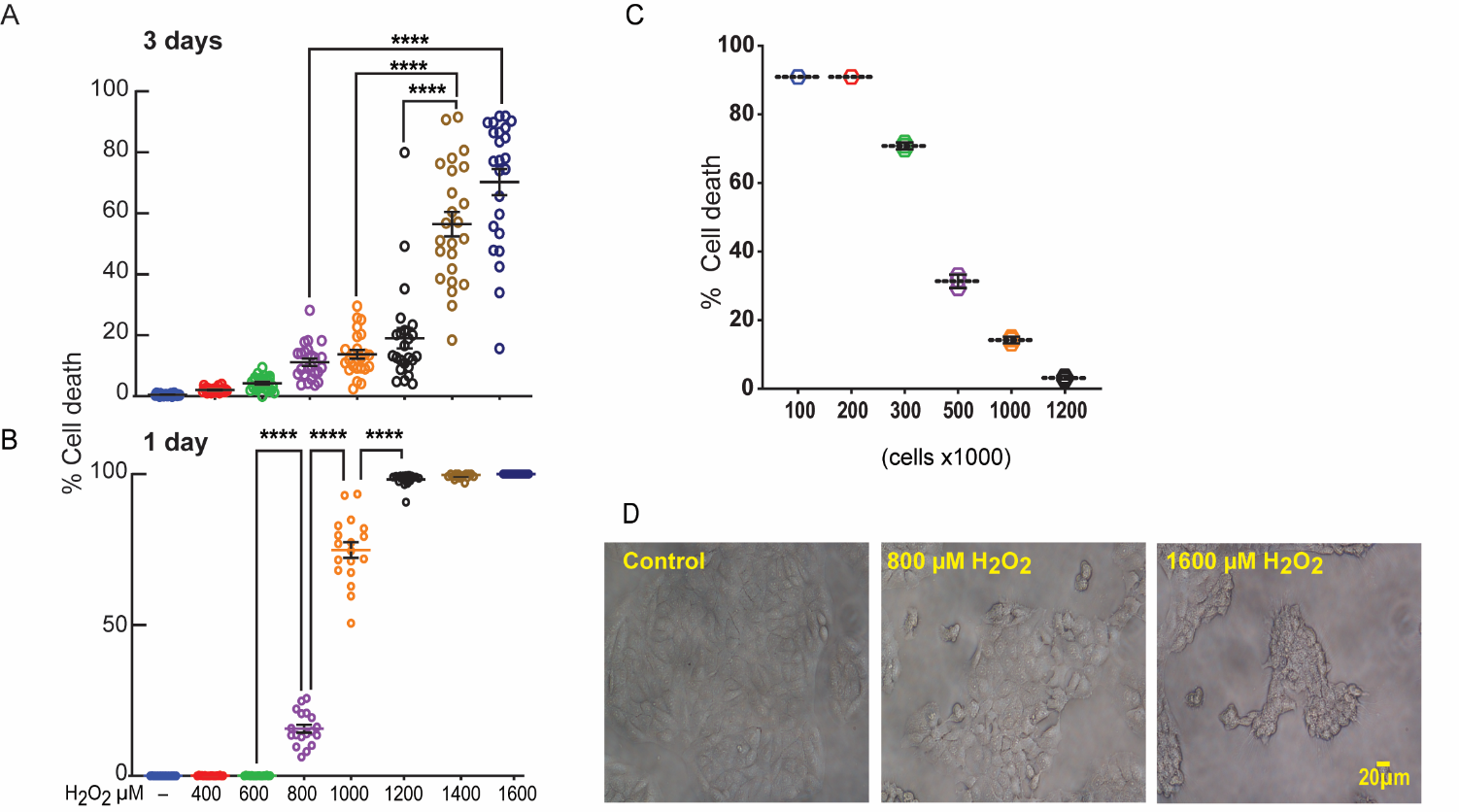
**

**Supplementary Figure 3.** **(A,B)** ABC cells were either grown for 3 days or overnight and were serum-starved for 8 h and then exposed to increasing concentrations of H_2_O_2_ (400 – 1600 µM). **(C)** ABC cells were grown in 24-well plates overnight (16 h) in 10% DMEM medium containing 10 ng/ml FGF-β at 100,000-1,200,000 cells per well. Cells were exposed to oxidative stress using 1200 µM H_2_O_2_ +TNF-α (10 ng/ml) in all conditions indicated in the figure and incubated 16 h at 37°C. Apoptotic cell death was detected by Hoechst staining. Images were captured by a Zeiss well plate scanner, and cell death was scored by an ImageJ program and then plotted. **(D)** Snapshots of live imaging of ABC cells exposed to UOS.

**
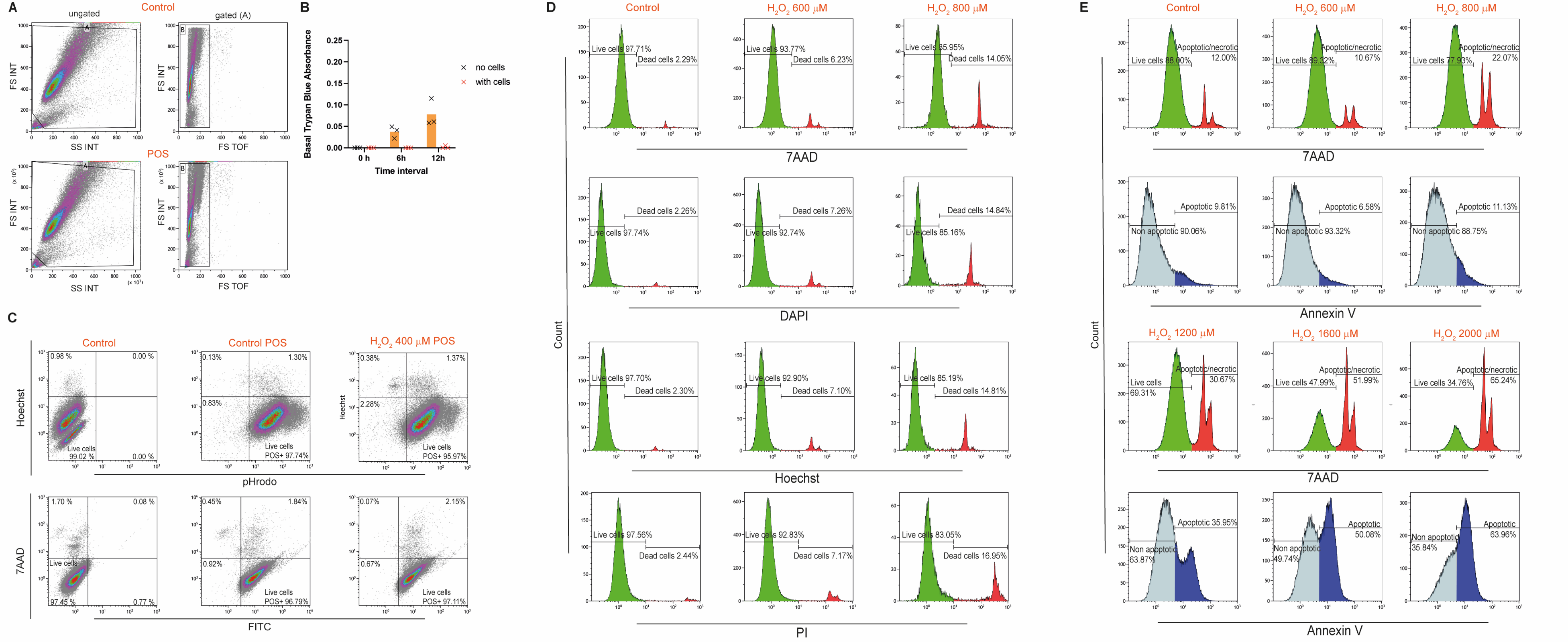
**

**
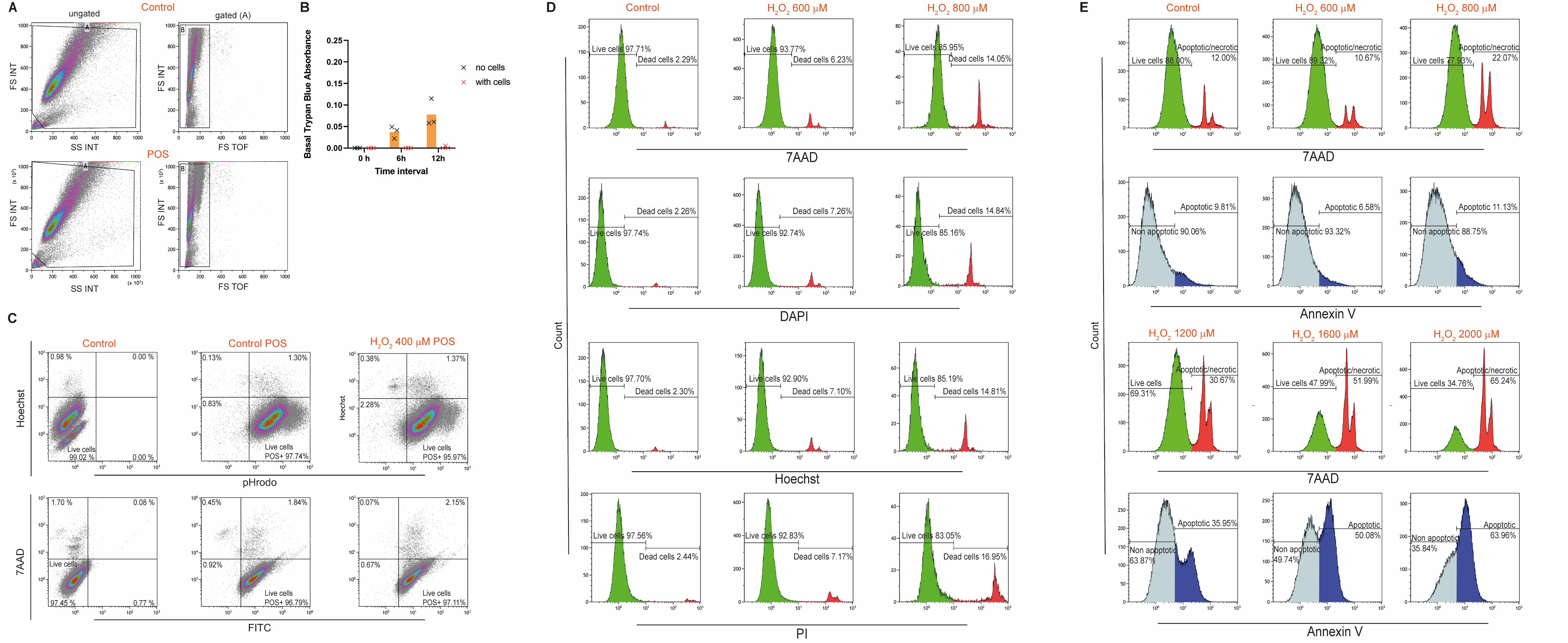
**

**
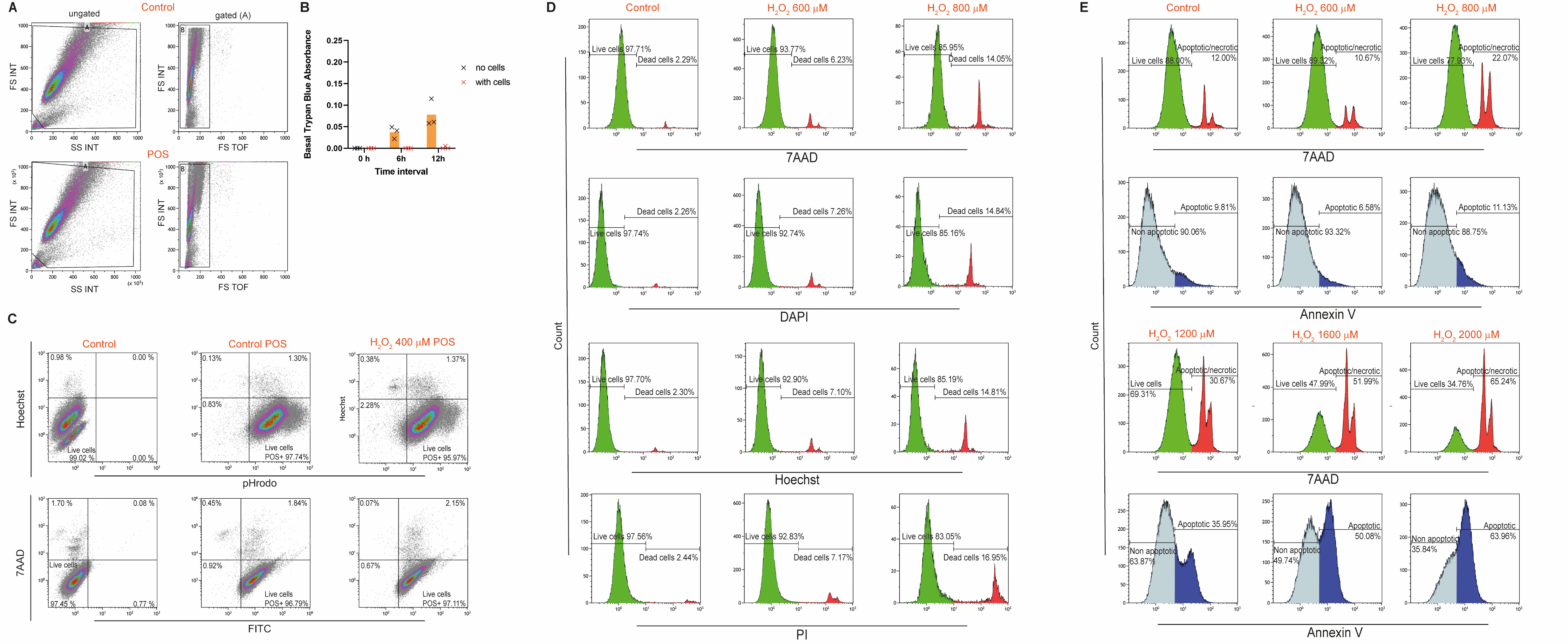
**

**Supplementary Figure 4.** **(A)** Flow cytometry gating strategies for control ABC cells and ABC cells exposed to FITC-POS. The plots on the left side show a forward scatter versus side scatter for 100,000 events in each condition. Gating strategy for removing debris (gate A) and doublets (gate B) is shown. **(B)** The basal flux was calculated by photometry measurements after subtracting the background and is expressed in absorbance at 560 nm. Plots of trypan blue concentration on the basal side, opposite the loading side, of control inserts without cells (black crosses) and with cells (red crosses) over time are shown. **(C)** Percentage of viable cells positive for POS. Confluent ABC-RPE cell cultures were challenged with pHrodo-POS or FITC-POS for 8 h and then with H_2_O_2_ for another 16 h, then cells were trypsinized, labeled with Hoechst (pHrodo cells) or 7AAD (FITC cells) and analyzed by flow cytometry. 100k events were analyzed in each condition. The graphs showed that independently of the fluorescent labeling of POS, a high percentage of cells incorporate POS, and this percentage is not affected in either case by a low H_2_O_2_ concentration. **(D)** ABC cells were plated and allowed to grow for 1 day until confluency and then were challenged with H_2_O_2_ for 16 h. Cells were trypsinized, labeled with different cell dyes (7AAD, DAPI, HOECHST, or PI), and analyzed by flow cytometry to determine UOS concentration-dependent apoptosis of ABC cells. 20k events were analyzed in each condition. We obtained similar results among all of them. **(E)** ABC cells were grown for 2 days, serum-starved for 8 h, and exposed to different OS concentrations for the next 16 h. Then cells were trypsinized, labeled, and analyzed by flow cytometry using an Apoptosis/Necrosis detection kit. Cell debris and doublets were excluded by gating (data not shown), and plots showing Annexin V or 7AAD signals were made (100k events per condition were assessed).

**
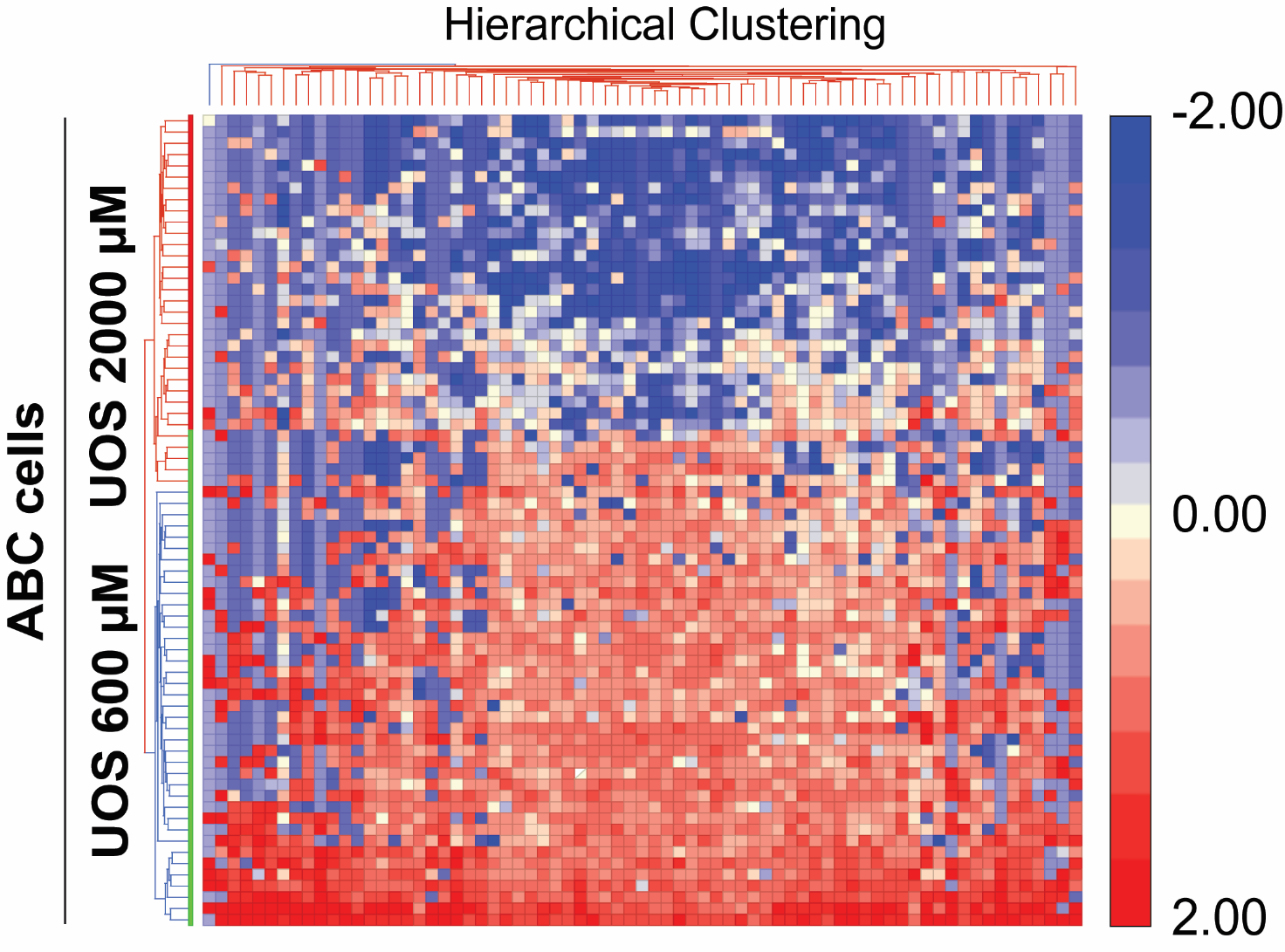
**

**Supplementary Figure 5.** Hierarchical clustering of statistically significant genes showed a marked downregulation of the majority of stress response and inflammatory genes with 2000 μM, signifying that higher concentrations of stress shut down the transcriptional machinery. Consequently, 600 μM H_2_O_2_ was used for all the later experiments.

**
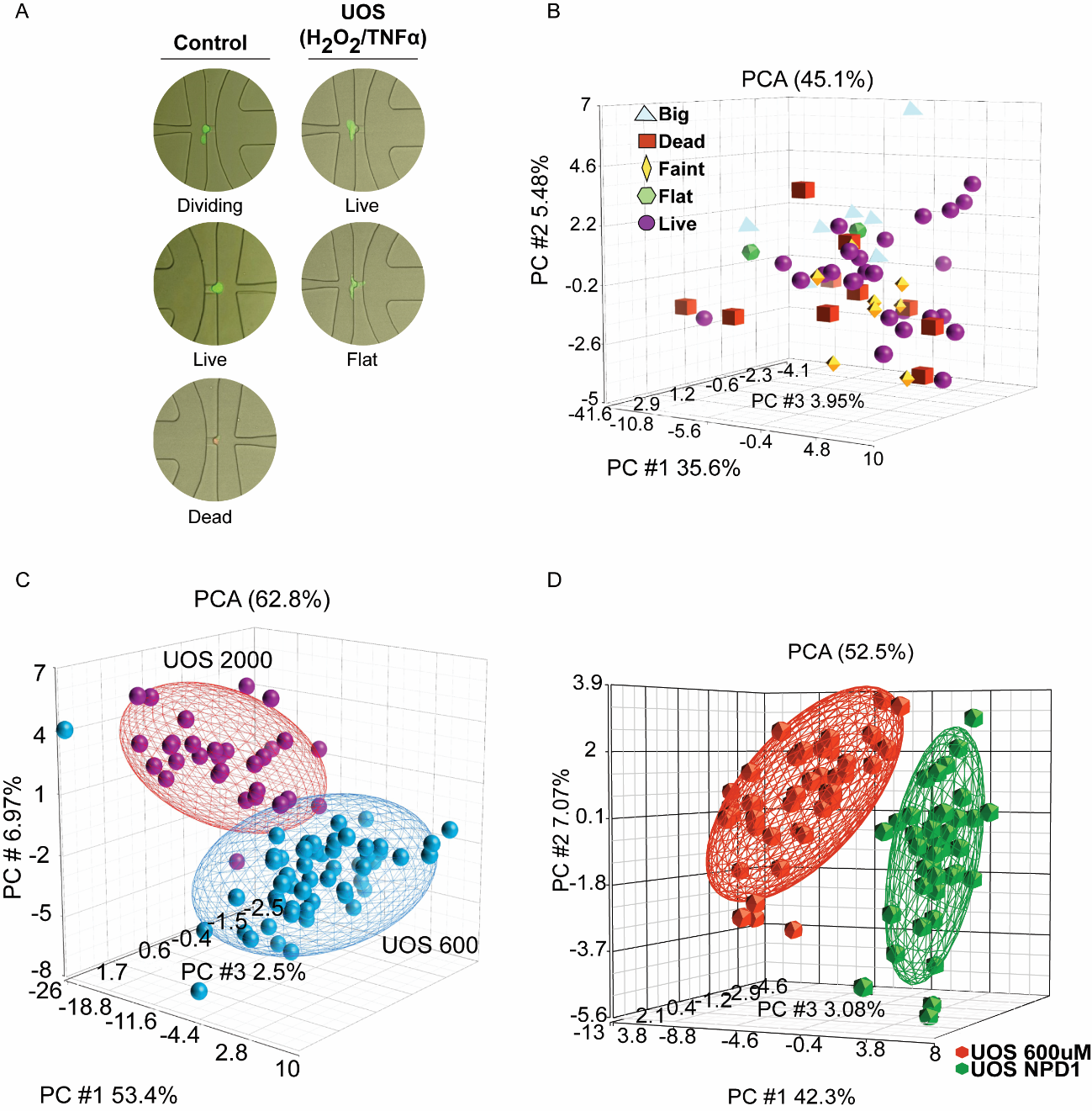
**

**Supplementary Figure 6.** ABC cells were treated with 600 μM or 2000 μM H_2_O_2_ for 6 h, followed by trypsinization. Single cells were then isolated, lysed, and pre-amplified in the C1™ Fluidigm platform. The final real-time amplification was performed using the Biomark™ system that utilizes high-definition optics for precise measurements (**Supplementary Figure 7A**). **(A)** Treatment with 600 μM H_2_O_2_ changes the morphology of captured cells (live cells positive to Calcein-AM and dead cells, positive to Ethidium Homodimer). Control cells were identified as live and dividing, while ABC cells subjected to the H_2_O_2_ were identified as live, flat, and dead. **(B)** PCA analysis did not show any specific clustering based on the cell morphological changes. **(C)** PCA shows two distinct groups when the gene expression data are compared in response to two different concentrations of oxidative stress (600 μM vs. 2000 μM). **(D)** PCA analysis shows a significant effect of NPD1 on the single-cell transcriptome when cells are challenged with oxidative stress.

**
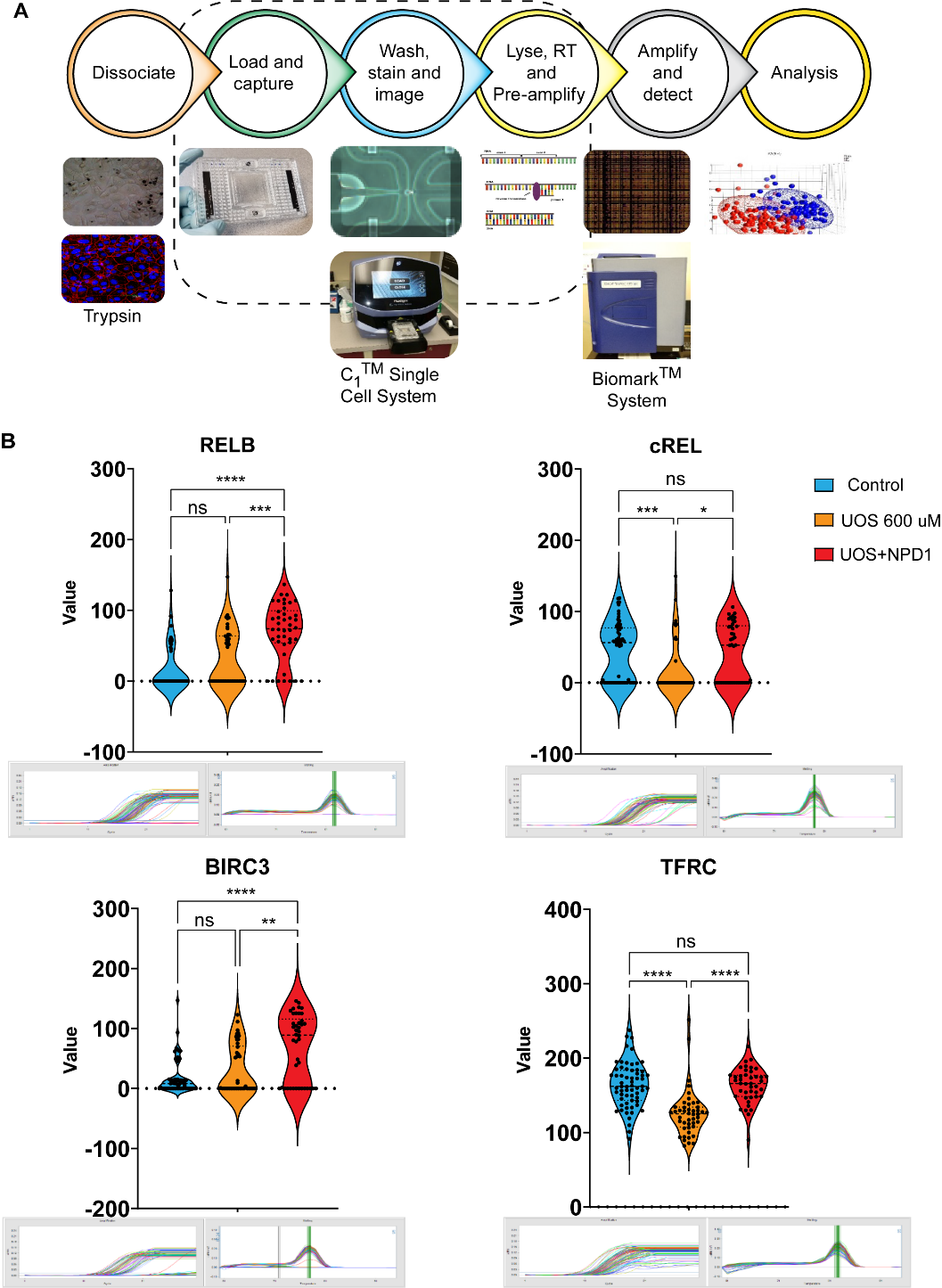
**

**Supplementary Figure 7.** **(A)** Single cell gene expression workflow. After induction of oxidative stress and lipid treatment, the cells are dissociated and loaded into the C1™ chip for isolation. Each single cell is lysed, and its mRNA transcripts are reverse transcribed into cDNA that will be amplified. The resulting cDNA is used for high throughput qPCR with the Biomark™, and the gene expression is analyzed for each individual cell. **(B)** Violin plots and box plots showing the expression of RELB, cREL, BIRC3, and the housekeeping gene TFRC for a population of single ABC cells. Respective melting curves, as well as real-time logarithmic graphs, are shown below for each gene. Y-axis demonstrates the relative expression of each gene. Each black dot on the right of the violin plots represents the expression of the gene for a single ABC cell.

**
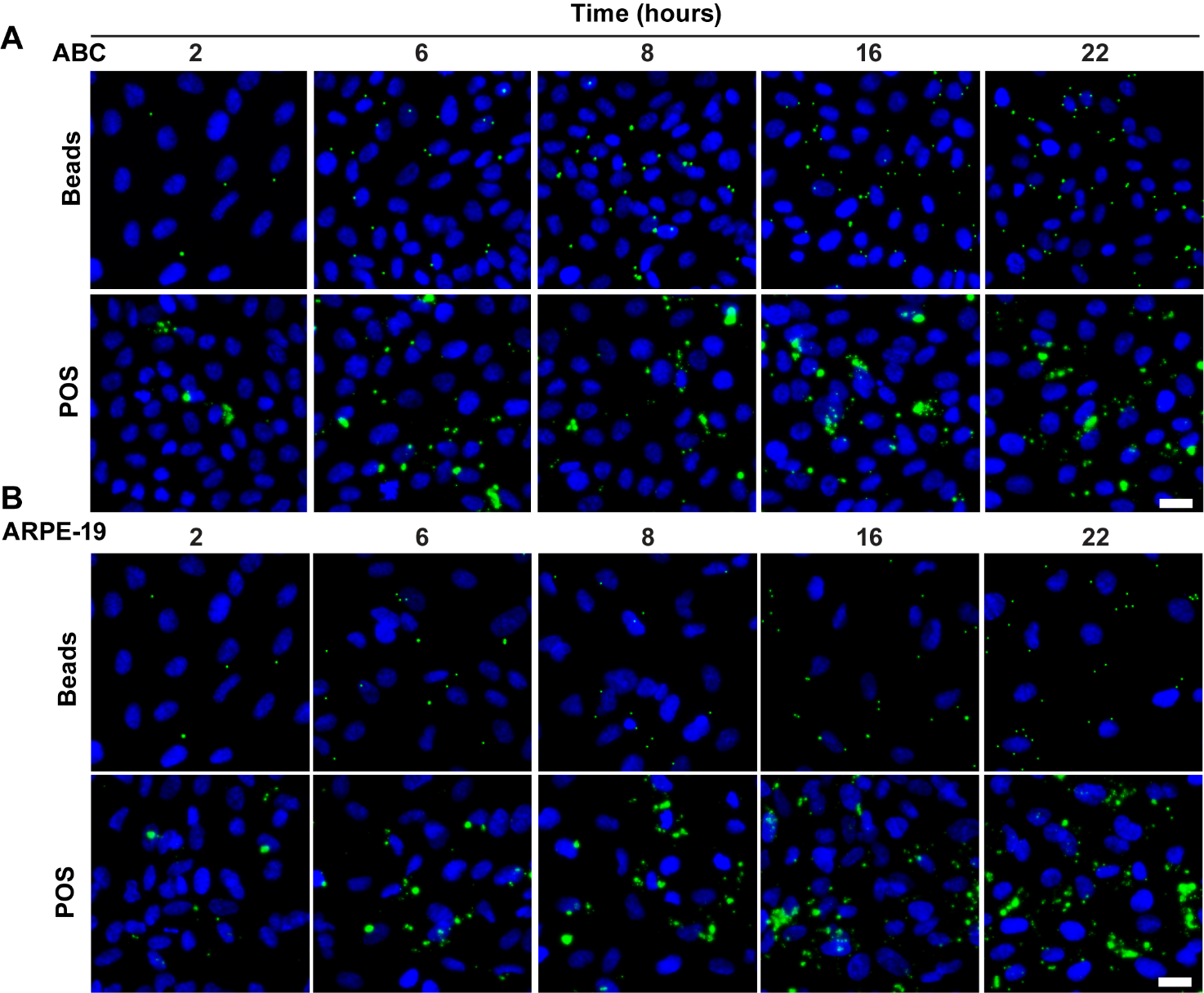
**

**Supplementary Figure 8.** **(A)** Rate of phagocytosis of fluorescent polystyrene microspheres (beads) or FITC-labeled POS by ABC cells. **(B)** ARPE-19 cells over time. Confocal microscopy was used to analyze bound and internalized POS or beads. Green fluorescence from the added material (beads or POS) and blue fluorescence from the nuclear staining Hoechst of the cells.

**
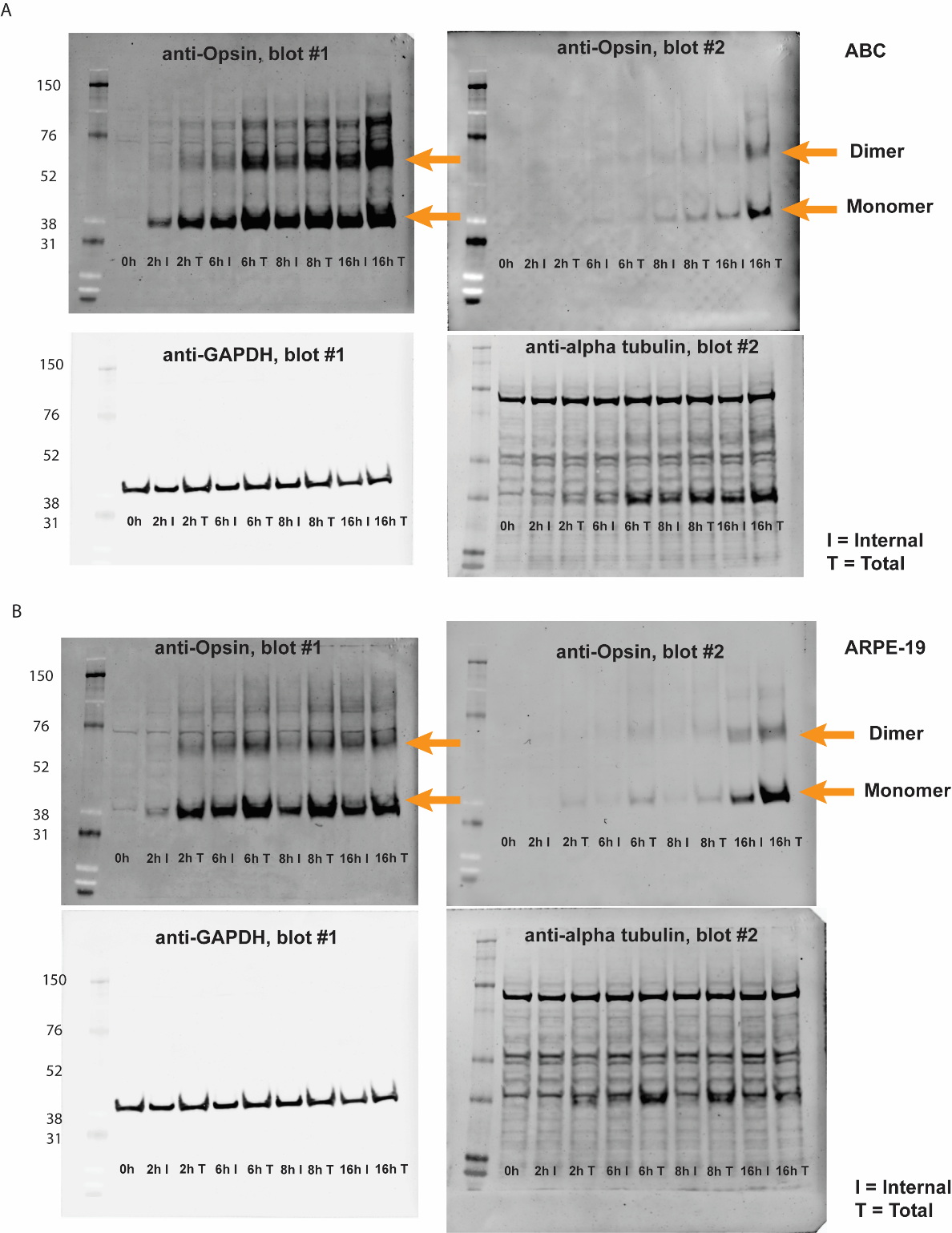
**

**Supplementary Figure 9.** ABC **(A)** and ARPE-19 **(B)** cells were incubated with unlabeled POS suspension for 0, 2, 6, 8, and 16 h and then evaluated for internalized and total (internalized+bound) POS. Analysis of phagocytosed POS content in samples was determined by SDS-PAGE electrophoresis and opsin immunoblotting. GAPDH was used as loading control for the first blot and alpha-tubulin was used as loading control for the technical replicate (blot #2). Molecular weights are in kDa. The dimer and monomer forms of the opsin (anti-rhodopsin) are labeled with arrows.

**
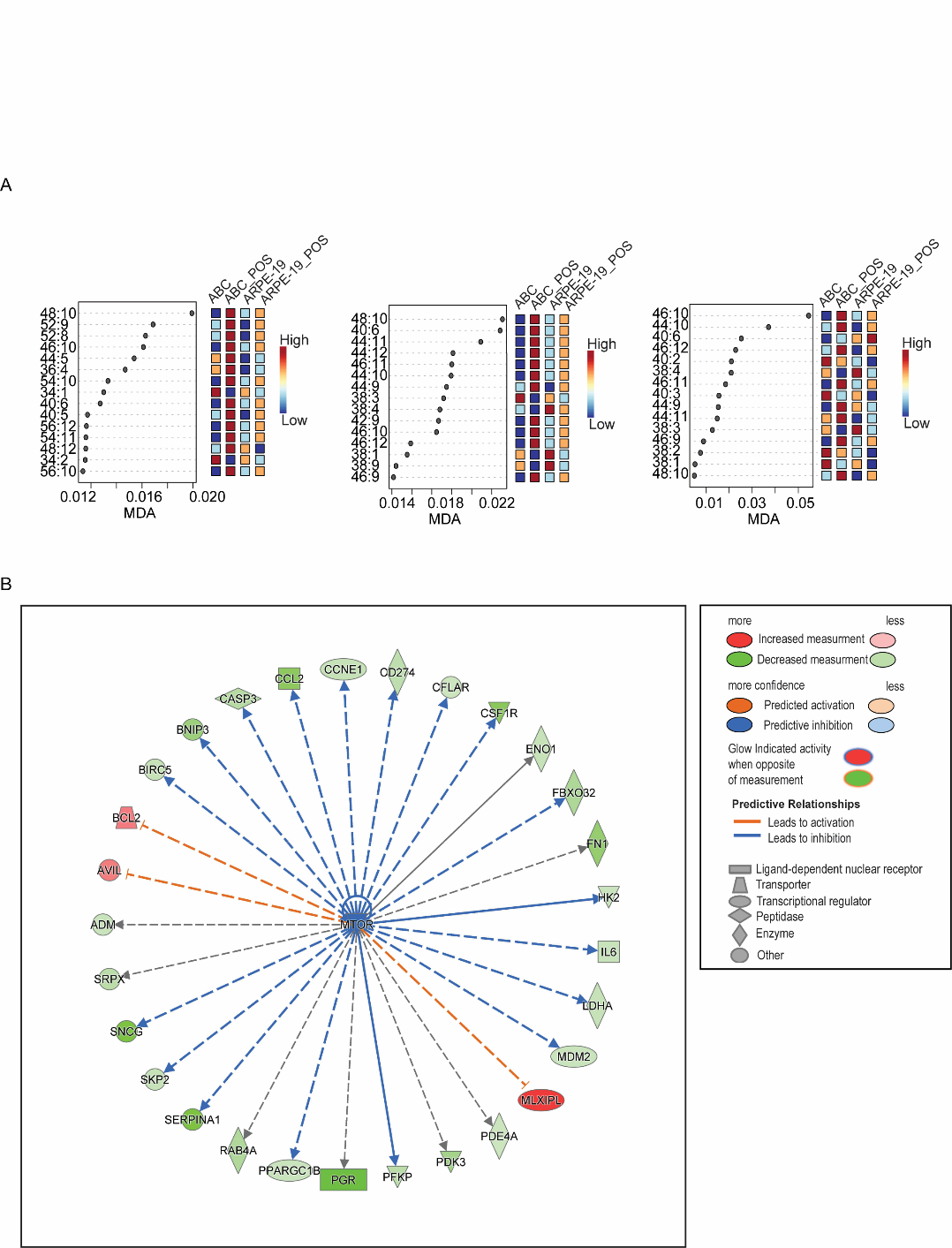
**

**Supplementary Figure 10.** Random forest analysis **(A)** were performed for PC, PE, and PS phospholipid species for ABC cells, and ARPE-19 cells with and without POS. The cells grown in 24-well chambers were fed with POS for 7-9 days and collected for analysis of the incorporation of phospholipid species (PC, PE, and PS). For random forest analysis, the variables (phospholipid species) are presented by descending importance. Mean Decrease Accuracy (MDA) shows, for each variable, how important it is in classifying the data. The MDA plot expresses how much accuracy the model losses by excluding each variable. The more the accuracy suffers, the more important the variable is for the successful classification. The higher the value of MDA is, the higher the importance of the variable in the model. The heatmap shows comparative assessment of each phospholipid (PLs) species for all four conditions (ABC ± POS and ARPE-19 ± POS). **(B)** The detailed IPA-predicted signaling pathways of mTOR for ABC and ARPE-19 cell comparison is shown. The blunt blue arrows represent inhibited interaction, blunt orange arrows represent activated interaction, and the red tip arrows represent activated interaction.

# Supplementary Tables

**Supplementary Table 1.** Primers used for RT-qPCR.

| **Target** | **Forward primer** | **Reverse primer** | **Design RefSeq** |
| --- | --- | --- | --- |
| ADIPOR1 | TGAACTGGGACCCCTGCTA | GGGCATGTTTGCTCTTCTTCA | NM_015999.3 |
| ADIPOR2 | ATGTTTGCCACCCCTCAGTA | GAACCCCTCCGAGATGACATA | NM_024551.2 |
| AIM2 | GCATCTGCAGCCATCAGAAA | GCCACCATCTGTTTCTGTTCA | NM_004833.1 |
| AKT1 | CACACACTCACCGAGAACC | TCGTGGGTCTGGAAAGAGTA | NM_005163.2 |
| APAF1 | AAGAGGCTAAAGACCGTCTCC | ACCCAAGAGTCCCAAACATCA | NM_013229.2 |
| ASCL1 | TGGTGCGAATGGACTTTGGAA | CTCCCAACGCCACTGACAA | NM_004316.3 |
| ATG10 | GTGATAGTTGGGAATGGAGACC | GTGACATCACAGACCCATTCTTA | NM_001131028.1 |
| ATG12 | CCCAGACCAAGAAGTTGGAAC | ACGCCTGAGACTTGCAGTAA | NM_004707.3 |
| ATG16L1 | GTCACGACCGGACTCTCAAA | TTGCAACTGGATCCTGCAAAC | NM_030803.6 |
| ATG16L2 | TTCGGGACCGTACGCAAAA | CAGCAGCTCAGCCTTCTCTAA | NM_033388.1 |
| ATG2A | GCTCAGCCTCGATCTGTACAA | CTCCAGCACCTCGTTCACA | NM_015104.2 |
| ATG2B | GGGGCTTTTGGAGTTAACCA | CGCTGGAACAGTGTAACTCA | NM_018036.5 |
| ATG3 | CCTCCCATGTGTTCAGTTCA | CTCCTTCTGCAACAGTCTCA | NM_022488.3 |
| ATG5 | GACCAGTTTTGGGCCATCAA | GGACGAAACAGCTTCTGAATGAA | NM_004849.2 |
| ATG7 | GCACCTTGGGTTGCAATGTA | GAGATCTTGGCATTGTCCACAAA | NM_001144912.1 |
| ATG9A | GCACCAGAAGAATGGCTTCA | GGTAGTGAAGGCAACCACAAA | NM_024085.3 |
| ATG9B | GAACCTGGACAGTTTCTTCAC | GGAAGACATCCTCCAGCAA | NM_173681.5 |
| ATIC | CAGTGGCTTCTCCAGGTGTAA | TTGGCTGCAGCTCTCAGTAA | NM_004044.6 |
| BAD | AGCAGGAGCAGCCAACCA | GTAGGAGCTGTGGCGACTCC | NM_004322.3 |
| BAX | GGGTTGTCGCCCTTTTCTAC | TCTTGGATCCAGCCCAACA | NM_004324.3 |
| BCL2 | ATGTGTGTGGAGAGCGTCAA | GTGCCGGTTCAGGTACTCA | NM_000633.2 |
| BCL2L10 | TGGTCCAGGCTTTTCTGTCA | GGCAGGTAGAAGCGGGTTA | NM_001306168.1 |
| BECN1 | GAGACCCAGGAGGAAGAGACTA | AATCTGCGAGAGACACCATCC | NM_003766.3 |
| BID | CCCTGCAGCTCAGGAACA | CTCCTTCTCCATGTCTCTAGGGTA | NM_197966.1 |
| BIRC2 | CTTCAGATACCACTGGAGAAGAAAA | GAAGAACTTTCTCCAGGTCCAAA | NM_001166.3 |
| BIRC3 | CAGACAGCCCAGGAGATGAAA | CTGAATGGTCTTCTCCAGGTTCA | NM_001165.4 |
| BIRC5 | GACCACCGCATCTCTACATTCA | CCAAGTCTGGCTCGTTCTCA | NM_001168.2 |
| CAMK2A | AACACCACCATCGAGGATGAA | CTTCAATCAGCTGCTCTGTCAC | NM_015981.3 |
| CASP1 | CATTTGAGCAGCCAGATGGTA | GTCCTGGGAAGAGGTAGAAACA | NM_001223.3 |
| CASP3 | AGGCCGACTTCTTGTATGCA | AACCAGGAGCCATCCTTTGAA | NM_004346.3 |
| CASP7 | TGACTTCCTCTTCGCCTATTCC | ACAAACCAGGAGCCTCTTCC | NM_033338.5 |
| CASP8 | GGAAATCTCCAAATGCAAACTGG | CAGGATGACCCTCTTCTCCAT | NM_001080124.1 |
| CCL5 | CCCTCGCTGTCATCCTCA | GGGCAATGTAGGCAAAGCA | NM_002985.2 |
| COL1A1 | CCCAAAGGATCTCCTGGTGAA | GCCAGGGCTTCCAGTCA | NM_000088.3 |
| CXCL12 | GCTGGTCCTCGTGCTGAC | GAATCGGCATGGGCATCTGTA | NM_199168.3 |
| CXCL2 | CCAAAGTGTGAAGGTGAAGTCC | CGATGCGGGGTTGAGACA | NM_002089.3 |
| DAPK1 | CCTTCTGGGCTCATTATCCAAC | CAGCCAGCAGCAATGAGTAA | NM_004938.2 |
| DAPK2 | GAGCATAGGCGTCATCACCTAC | TGTTTCCTGCTTCGTGTCTCC | NM_014326.3 |
| DKK3 | AATGGGACCATCTGTGACAACC | GCAAAGCTCGCCCTCCA | NM_015881.5 |
| DLG4 | AGCTGGAGCAGGAGTTCAC | ACACGCTTCACCTTGTGGTA | NM_001365.3 |
| DTX3L | GCTGTGGATTCCTGTCTCC | ACTAGGGCACAGTGTGGTTA | NM_138287.3 |
| EGFR | GCAGTGACTTTCTCAGCAACA | TTGGGACAGCTTGGATCACA | NM_005228.3 |
| ELK1 | GGGCCTTGCGGTACTACTA | GGGTAGGACACAAACTTGTAGAC | NM_001114123.2 |
| EN1 | TGGGTGTACTGCACACGTTA | CTGCTCGGCCGTGAAC | NM_001426.3 |
| FADD | CTGACCGAGCTCAAGTTCCTA | GCAGCATGGAGAAGAGGTCTA | NM_003824.3 |
| FAS | GCATCTGGACCCTCCTACC | CCTTGGAGTTGATGTCAGTCAC | NM_000043.4 |
| FOXG1 | GCCAGCAGCACTTTGAGTTA | TGAGTCAACACGGAGCTGTA | NM_005249.3 |
| FOXO1 | GGTGTCAGGCTGAGGGTTA | TTCTCTCAGTTCCTGCTGTCA | NM_002015.3 |
| FOXO3 | CGCTCTCTCCGCTCGAA | TTTGCAGGGGCCACGTA | NM_001455.3 |
| GAD1 | ATCCTGGTTGACTGCAGAGAC | CCAGTGGAGAGCTGGTTGAA | NM_000817.2 |
| GAD2 | CTGCTCCAAAGTGGATGTCAAC | AAAGTGGGCCTTTCTCCATCA | NM_000818.2 |
| GAPDH | GAACGGGAAGCTTGTCATCAA | ATCGCCCCACTTGATTTTGG | NM_002046.4 |
| GFAP | GCCAGTTGCAGTCCTTGAC | GCGCATCTGCCTCTCCA | NM_002055.3 |
| GRIA1 | CGCTCCACGTGATTGAAATGAA | TGGCTGCAGGGACAAACTTA | NM_000827.3 |
| GRIA2 | TGGAATGGGATGGTTGGAGAA | CACCTCTTCTCTCACAAGGGTA | NM_001083619.1 |
| GRIN1 | GGCAACACCAACATCTGGAA | CCATCCGCATACTTGGAAGAC | NM_021569.3 |
| GRIN2A | GACCGGCCTCAGTGACAA | AGGCACTGTCCCAAATCGAA | NM_000833.3 |
| HES5 | CACTGTGGCCGTGGAG | GCTCGATGCTGCTGTTGAT | NM_001010926.3 |
| HOXA2 | GCCAAGAAAACCGCACTTCT | GCCATCGGCGATTTCCAG | NM_006735.3 |
| HOXA3 | GCAGAAAACCAGCAGCTCCA | CGCGCGCTTGGACGAA | NM_030661.4 |
| HOXB2 | GCACGGCTTACACCAACAC | GGCCGGCACAGGTACTTATTA | NM_002145.3 |
| IL17A | ACTACAACCGATCCACCTCAC | ACTTTGCCTCCCAGATCACA | NM_002190.2 |
| IL18 | ACCAAGGAAATCGGCCTCTA | ACCTCTAGGCTGGCTATCTTTA | NM_001562.3 |
| IL1A | CTGCCCAAGATGAAGACCAA | AGGAGGTTGGTCTCACTACC | NM_000575.3 |
| IL1B | GACCTGAGCACCTTCTTTCC | CGTGCACATAAGCCTCGTTA | NM_000576.2 |
| IL23A | TCACAGAAGCTCTGCACAC | TCCACACTGGATATGGGGAA | NM_016584.2 |
| IL6 | AGAGCTGTGCAGATGAGTACAA | GTTGGGTCAGGGGTGGTTA | NM_000600.3 |
| IL8 | ACACTGCGCCAACACAGAAA | CAGTTTTCCTTGGGGTCCAGAC | NM_000584.2 |
| IRAK1 | AGTGAAGCAGAGCTTCCTGAC | TGAGCACAGTAGCCAGCAAA | NM_001025243.1 |
| KCNC1 | GCTCTTCGAGGACCCGTAC | AGACCAGGATGAAGAAGAGGGAA | NM_001112741.1 |
| KCNC2 | CTCGTCCACTCGGAAACCA | ATCTTGCCCATCTCTGTGAC | NM_153748.1 |
| MAP1LC3A | GTCCCGGACCATGTCAACA | GGGCGTGGACACACTCA | NM_181509.1 |
| MAP1LC3B | AGTACAGCGGGAGAAACACA | CTTTAAGCCGGAAGGCAGAA | NM_022818.4 |
| MAP1LC3C | TGCTGGAATCCGGGCAAA | GGAACGTCTCCCTGGGGTA | NM_001004343.2 |
| MAP2 | CAACGGAGAGCTGACCTCA | CTACAGCCTCAGCAGTGACTA | NM_001039538.1 |
| MAPK1 | TTGGTACAGGGCTCCAGAAA | TCTGCCAGAATGCAGCCTA | NM_002745.4 |
| MAPK3 | GCTACACGCAGTTGCAGTACA | GTCTTGCGCACGTGGTCATA | NM_002746.2 |
| MBP | CAGAGGCACGGATCCAAGTA | CTCTGTGCCTTGGGAGGAA | NM_001025081.1 |
| MEFV | GTCTGAGTCAGGAGCACCAA | TCAGCTTCTTCAGATGCTCCA | NM_001198536.1 |
| MFRP | CCACCTACCTGGCCTTCAA | ATCCACTGCACACCCTTACA | NM_031433.2 |
| MFRP | CCACCTACCTGGCCTTCAA | ATCCACTGCACACCCTTACA | NM_031433.2 |
| MYD88 | CTGCAGAGCAAGGAATGTGAC | TGCTGGGGAACTCTTTCTTCA | NM_001172567.1 |
| NAIP | GCTTCATTGTCTCCGAGTCC | GGAAACCTCCACTGATTGCTAC | NM_004536.2 |
| NCAM1 | CTCCCAGTCCATGTACCTTGAA | GGTTCCCCTCCCAAGTGTAC | NM_181351.3 |
| NEUROD1 | GGCCCCAGGGTTATGAGACTA | ATCAGCCCACTCTCGCTGTA | NM_002500.4 |
| NEUROD2 | CCACTCGGAGAATCTCTTGTCTTA | AACGCATTGAGCTCCTCGTA | NM_006160.3 |
| NFE2L2 | GAGCAAGTTTGGGAGGAGCTA | GGTAGTCTCAACCAGCTTGTCA | NM_001145412.1 |
| NFKB1 | CTACCTGGTGCCTCTAGTGAAA | ACCTTTGCTGGTCCCACATA | NM_001165412.1 |
| NGF | ACCAAGGGAGCAGCTTTCTA | AGTGTGGTTCCGCCTGTA | NM_002506.2 |
| NKX2-2 | ACCGAGGGCCTTCAGTAC | CCTTGTCATTGTCCGGTGAC | NM_002509.2 |
| NKX6-1 | ACGCCTGGCCTGTACC | GAAGATCTGCTGTCCGGAAAA | NM_006168.2 |
| NLRC4 | CTTGTCTGCAAATGCAGTGAAAA | TCAAGAATGCTCAGTTTGACCAA | NM_021209.4 |
| NLRC5 | TGTCCAGCACTTAGCTACCA | GCTGAGCTGATGCTATTCCC | NM_032206.4 |
| NLRP3 | TCGGAGACAAGGGGATCAAA | CGTGAGGTTGCAGTTGTCTA | NM_001079821.2 |
| NLRX1 | TGCATGACCAGTGCCAAATTAC | CTCCATTAGCACGGCAACAC | NM_024618.2 |
| NOD1 | GCAGATGCGTTACAGAGCAA | ACTTTGGCCTCCTCTGGTTTTA | NM_006092.2 |
| NOD2 | AAATCAGGTTGCCGATCTTCA | GCCAATCCATTCGCTTTCAC | NM_022162.2 |
| NR4A2 | TGGCTGTTGGGATGGTCAAA | TCTTCGGTTTCGAGGGCAAA | NM_006186.3 |
| OLIG2 | CGGAGCGAGCTCCTCAAA | ATGGCCCCAGGGGAAGATA | NM_005806.2 |
| PARP9 | TGTCCAGGGCCACATTGAA | TGCCACAGGTCCAACTGTAA | NM_001146104.1 |
| PHB | GCGTGGTGAACTCTGCCTTA | CCACAATGTCCTGCACTCCA | NM_002634.2 |
| PLP1 | TCCATGCCTTCCAGTATGTCA | GGTGGTGTAGAAGCCCTCA | NM_001128834.2 |
| POU3F2 | CGGATCAAACTGGGATTTACCC | CGAGAACACGTTGCCATACA | NM_005604.3 |
| PYCARD | AGTTTCACACCAGCCTGGAA | GGTAGGACTGGGACTCCCTTA | NM_013258.3 |
| REL | TTCCTCCTGTTGTCTCGAACC | TCCACAATTCTTGTTTACACGACAAA | NM_002908.2 |
| RELA | CGGTGGGGATGAGATCTTCCTA | GCCTGGTCCCGTGAAATACA | NM_021975.3 |
| RELB | TGCTTTCCGAGCCCGTCTA | CGGCCCGCTTTCCTTGTTAA | NM_006509.2 |
| RIPK1 | AGTACCTTCAAGCCGGTCAAA | CAGGACCCATCCCAAGTCC | NM_003804.3 |
| RIPK2 | GGGCCAGTATCAAGCACGATATA | GGCTGTTTTCTGGATAACACTTCC | NM_003821.5 |
| RNF146 | GAGCTTCTGTTCAGCCCCTA | GGTGTTGCAGGGCTTGTTAA | NM_001242844.1 |
| SIRT1 | ACAAAGTTGACTGTGAAGCTGTAC | GTTCATCAGCTGGGCACCTA | NM_012238.4 |
| SLC17A6 | TGGGGCTACATCATCACTCA | GAAGTATGGCAGCTCCGAAA | NM_020346.2 |
| SLC17A7 | GCCATCTCCTTCCTGGTCCTA | GGCTATGTCCAGGTGGTTCAC | NM_020309.3 |
| SLC6A12 | TGCTACCTGATAGGGCTTTTCC | GCCACTGGAAGCATAGTAGTCA | NM_001122848.1 |
| SOD1 | GTGAAGGTGTGGGGAAGCATTA | TGAGGACCTGCACTGGTACA | NM_000454.4 |
| SOD2 | AGGAACGGGGACACTTACAA | TCAATCCCCAGCAGTGGAATA | NM_001024465.1 |
| SOX2 | CATGAAGGAGCACCCGGATTA | CGGGCAGCGTGTACTTATCC | NM_003106.2 |
| SQSTM1 | AGGAAGCTGCCTTGTACCC | TCTGGGAGAGGGACTCAATCA | NM_001142298.1 |
| STAT1 | ATGCTGGCACCAGAACGAA | GCTGGCACAATTGGGTTTCAA | NM_007315.3 |
| SYN1 | GCAAGGACGGAAGGGATCA | TGTCTTCATCCTGGTGGTCAC | NM_133499.2 |
| TAB1 | TGATGTCGGAGGGGTTGTAC | CAGTGTCAATCATCGCAGCAA | NM_006116.2 |
| TAB2 | CTGATGATGCTGCCTACACA | CAAGTTCTCTTTGAAGTCGTTCC | NM_015093.4 |
| TBP | TGCCCGAAACGCCGAATATA | CGTGGTTCGTGGCTCTCTTA | NM_001172085.1 |
| TFRC | CCAGTTGGAGTGCTGGAGAC | AGCCTTTAAATGCAGGGACGAA | NM_001128148.1 |
| TGFB1 | CGTCTGCTGAGGCTCAAGTTA | TCGCCAGGAATTGTTGCTGTA | NM_000660.4 |
| TH | GATTGCTGAGATCGCCTTCC | ATCTCCTCGGCGGTGTAC | NM_000360.3 |
| THY1 | TCAGCATCGCTCTCCTGCTA | TCCACTAGGCAGGCCGTTA | NM_006288.3 |
| TLR2 | GCAAATCCTGAGAGTGGGAA | GTTCCTCAAGGAAGGTAAGTCC | NM_003264.3 |
| TLR4 | ATTGACAGGAAACCCCATCCA | ACAGCCACCAGCTTCTGTAA | NM_138554.4 |
| TNF | CTTCTCGAACCCCGAGTGAC | ACTGGAGCTGCCCCTCA | NM_000594.3 |
| TNFSF14 | ACTTGCCACCTGAGTCACA | GGTCCTTCAACCTCAGAGGAA | NM_003807.3 |
| TRAF1 | ACCGTCAGCCTCTTCTCC | TCCAGTGCCATCTCCATTCA | NM_001190945.1 |
| TRAF2 | TGCGGCAAGAAGAAGATCCC | AATCTGCAAGGGACTCGACAC | NM_021138.3 |
| TRAF3 | TGTGCAGCCCGAAGCA | ATGCTCTCTTGACACGCTGTA | NM_003300.3 |
| TRAF5 | TGTCCTGAAGCTGAGCAAGAC | GCTGCAGGTTCCTCCGTTTA | NM_004619.3 |
| TRAF6 | TGGCAAATGTCATCTGTGAATAC | GCTGTAGGGCAGTCTAGATCA | NM_004620.2 |
| TUBB3 | GAGCGGATCAGCGTCTACTA | GGTTCCAGGTCCACCAGAA | NM_006086.2 |
| XIAP | CAGCTTGCAAGAGCTGGATTT | CTTCCAATCAGTTAGCCCTCCT | NM_001167.3 |
